# Development and acceptability of a patient decision aid for people with degenerative cervical myelopathy: an international mixed-methods study

**DOI:** 10.1136/bmjopen-2025-106337

**Published:** 2026-04-03

**Authors:** Andrew R Gamble, David B Anderson, Marnee J McKay, Benjamin Davies, Sophie Macpherson, James Van Gelder, Tammy Hoffmann, Kirsten McCaffery, Samuel X Stevens, Carlo Ammendolia, Rohil V Chauhan, Carl M Zipser, Timothy F Boerger, Lindsay A Tetreault, Michael G Fehlings, Elanor Dustan, Carolyn Nugent, Helen Holmgren, Andreas K Demetriades, Justin M Lantz, Rana Dhillon, Chris G Maher, Joshua R Zadro

**Affiliations:** 1 School of Health Sciences, Faculty of Medicine and Health, The University of Sydney, Sydney, New South Wales, Australia; 2 Sydney Musculoskeletal Health and Institute for Musculoskeletal Health, Sydney School of Public Health, Faculty of Medicine and Health, The University of Sydney, Sydney, New South Wales, Australia; 3 Discipline of Neurosurgery, Cambridge University Hospital, University of Cambridge, England, UK; 4 Discipline of Neurosurgery, Concord Repatriation General Hospital, Sydney, New South Wales, Australia; 5 Medicine and Health, University of New South Wales, Sydney, New South Wales, Australia; 6 Institute for Evidence-Based Healthcare, Faculty of Health Sciences and Medicine, Bond University, Gold Coast, Queensland, Australia; 7 Sydney Health Literacy Lab, School of Public Health, Faculty of Medicine and Health, The University of Sydney, Sydney, New South Wales, Australia; 8 School of Medicine, Faculty of Medicine and Health, The University of Sydney, Sydney, New South Wales, Australia; 9 Department of Medical Oncology, Queen’s University, Kingston, Ontario, Canada; 10 Department of Surgery, Faculty of Medicine, University of Toronto, Toronto, Ontario, Canada; 11 AUT Active Living and Rehabilitation Research Centre, Faculty of Health and Environmental Sciences, Auckland University of Technology, Auckland, New Zealand; 12 Spinal Cord Injury Center, Balgrist University Hospital Zurich, University of Zurich, Zürich, Switzerland; 13 Joint Department of Biomedical Engineering, Marquette University and The Medical College of Wisconsin, Milwaukee, WI, USA; 14 Department of Neurology, Mass General Brigham, Boston, MA, USA; 15 Division of Neurosurgery and Spine Program, University of Toronto and Krembil Neuroscience Centre, Toronto Western Hospital, University Health Network, University of Toronto, Toronto, Ontario, Canada; 16 Nottingham University Hospitals, NHS Trust, Nottingham, UK; 17 Myelopathy.org, University of Cambridge, Cambridge, UK; 18 Edinburgh Spinal Surgery Outcomes Study Group, Department of Neurosurgery, Royal Infirmary Edinburgh, Scotland, UK; 19 Division of Biokinesiology and Physical Therapy, University of Southern California, Los Angeles, CA, USA; 20 Department of Surgery, University of Melbourne, Melbourne, Victoria, Australia; 21 Department of Neurosurgery, St Vincent’s Hospital, Melbourne, Victoria, Australia

**Keywords:** Orthopedics, Spine, NEUROSURGERY

## Abstract

**Abstract:**

**Objectives:**

To develop and user-test a patient decision aid for people diagnosed with degenerative cervical myelopathy and who are considering surgery.

**Design:**

Mixed-methods study describing the development of a patient decision aid.

**Setting:**

A draft decision aid was developed by a multidisciplinary steering group (including study authors with degenerative cervical myelopathy, health professionals and researchers) informed by the best available evidence, authorship consensus and existing patient decision aids.

**Participants:**

Patient-participants and health professional-participants who manage people with degenerative cervical myelopathy were recruited through social media and the steering group’s research and practice network. Quantitative questionnaires were used to gather baseline data, descriptive feedback, refine the decision aid and assess its acceptability. Qualitative semi-structured interviews were conducted online to gather feedback on the decision aid and were analysed using reflexive thematic analysis.

**Results:**

We conducted 32 interviews: 19 patient-participants and 13 health professional-participants who manage people with degenerative cervical myelopathy (neurosurgeons, neurologists, physiotherapists, orthopaedic surgeons, general practitioners, rehabilitation and pain specialists and consultant occupational physicians and chiropractors). Participants were from 10 countries (Australia, Canada, Cyprus, Germany, Ireland, New Zealand, Sweden, Switzerland, United Kingdom and USA). Most participants rated the decision aid’s acceptability as good-to-excellent and agreed with most aspects of the decision aid (eg, defining degenerative cervical myelopathy, management recommendations, potential benefits and harms, questions to consider asking a health professional).

**Conclusion:**

Our patient decision aid was rated as an acceptable tool by both health professional-participants who treat degenerative cervical myelopathy and patient-participants with lived experience of degenerative cervical myelopathy. This decision aid can be used by clinicians and people with degenerative cervical myelopathy to help with shared decision making following a diagnosis of degenerative cervical myelopathy. A study testing the potential benefits of this decision aid in a clinical setting is recommended.

**STRENGTHS AND LIMITATIONS OF THIS STUDY**

- We developed a decision aid that satisfies the International Patient Decision Aid Standards criteria and used mixed methods to evaluate acceptability of the decision aid.
- One-on-one interviews with patient-participants from diverse education levels and employment status, and health professional-participants from various professions allowed for in-depth feedback to be gathered on the decision aid.
- Acceptability of the decision aid in different countries may be limited by the number of interviews with participants from each country and being presented in English only.
- Most patient-participants who volunteered to participate were female and most health professional-participants interviewed were male.
- Data on benefits and harms in the decision aid were based on the only randomised controlled trial comparing non-surgical and surgical management for people with degenerative cervical myelopathy which was conducted at a single centre.

## Introduction

 Degenerative cervical myelopathy (DCM) is the most common cause of spinal cord dysfunction in adults, with an estimated prevalence of 2.2% in the general population and up to 5% in people over the age of 40.^1^,^4^ DCM can lead to a wide range of symptoms including upper and lower extremity sensory changes, gait impairment, clumsiness of the hands and bladder/bowel dysfunction.^5^,^7^ Subtle^8^ and diverse symptoms of DCM can mimic other conditions and may contribute to misdiagnosis.^1 9^ The quality of life of people living with DCM is worse than people living with other diseases such as diabetes or some cancers.^10^

People with DCM often experience long delays before receiving a diagnosis which can impact management recommendations and their quality of life.^11^ A diagnosis of DCM takes on average 2.2 years, but can take up to 9 years after an initial visit with a health professional.^1^ Delayed diagnosis is likely due to a lack of awareness of the condition among clinicians and the community.^12 13^ Earlier diagnosis and treatment models have been associated with improved quality of life and cost effectiveness.^14^

Clinical practice guidelines use the modified Japanese Orthopaedic Association (mJOA) scale to categorise DCM severity and guide management.^15^ The mJOA is an 18-point scale categorising the severity of DCM into non-myelopathic (18), mild (15–17), moderate (12–14) or severe (0–11).^15 16^ Guidelines suggest non-surgical management for people who are non-myelopathic (on the mJOA scale) and surgical management for people with moderate and severe DCM, but there is uncertainty about management for people with mild DCM.^17^ Only one randomised controlled trial (RCT) has been conducted comparing non-surgical and surgical management of people with mild-to-moderate DCM.^18^ The 10-year follow-up showed on average, no significant between-group differences in neurological function using mJOA scores at a single centre.^19^ However, outcomes following surgical decompression in this study differ from some other studies which may be due to surgical technique.^17^

An evidence-based resource is needed to guide management decisions for people with mild DCM given the uncertainty on the most appropriate management. One potential solution is a patient decision aid for people with mild DCM comparing non-surgical and surgical management.^20^ Patient decision aids outline expected benefits and risks of management options by providing evidence-based information.^21^ Patient decision aids aim to better align treatment choices with patient’s personal values and in many cases, promote multiprofessional involvement in treatment decisions.^22^ A patient decision aid could promote shared decision making for people with DCM and increase awareness of the condition for timely diagnosis and intervention.^13^

The aim of this study was to develop and user-test a patient decision aid for people with DCM to guide appropriate management.

## Methods

### Initial design of the decision aid

We developed a patient decision aid informed by the International Patient Decision Aid Standards (IPDAS) checklist and Collaboration Evidence Update 2.0.^23^ A multidisciplinary steering group (study authors) was assembled, including those with lived experience of DCM (CN, HH) physiotherapists, physicians and a surgeon who are topic experts on DCM (ARG, DBA, BD, SS and JVG), as well as patient decision aid and shared decision-making experts (ARG, JZ, DBA, MJM, TH, KM). The first draft of the decision aid was informed by the design of previous decision aids^24^,^26^ developed by some authors in the steering group (ARG, JZ, DBA, MJM, CM, SM, TH, KM). Key features adapted from these decision aids included outlining the condition and management options, using icon arrays to present potential benefits and harms, and providing questions to consider when talking to a health professional. Decision science evidence suggests these features can improve patient decision-making.^27^,^30^

To inform the content in the decision aid, a literature search (online supplemental file 1) was conducted based on a search strategy of a previous Cochrane review.^31^ Author consensus informed by the evidence hierarchy^32 33^ determined the information included. Diagnosis and management recommendations were informed by the most recent clinical practice guidelines for people with mild, moderate and severe DCM.^17^ Benefits and harms statistics were informed by various sources of evidence, including 10-year follow-up data^19^ from the only RCT comparing non-surgical and surgical management^18^ for people with mild-moderate mJOA scores, recent systematic reviews,^34^,^36^ a large observational study^37^ and the only study examining physiotherapy management for people with mild DCM via a web-based survey.^4^ We included additional statistics to the RCT to provide more information and context to aid decision making.^434^,^37^ The study authors provided feedback on the first draft of the decision aid before we began semi-structured interviews.

### Recruitment

Participants were recruited through social media (Myelopathy.org), snowballing, and via the steering group’s research and clinical practice network. All participants met the inclusion criteria (table 1) and provided consent to participate in the study by checking a box confirming they read the participant information sheet and consent form.

**Table 1:** Inclusion and exclusion criteria

	Inclusion criteria*
Patient-participants	18 years old or older DCM diagnosis verified by a health professional (eg, general practitioner) and an MRI scan Score of 17 or less on the mJOA scale Able to understand written and verbal English
Health professional-participants	Involved in the management of DCM Review ≥5 patients per year with DCM Able to understand written and verbal English

The mJOA is an 18-point scale categorising the severity of DCM into non-myelopathic (18), mild (15–17), moderate (12–14) or severe (0–11).^15 16^

* Potential participants were excluded if they did not meet the above inclusion criteria.

DCM, degenerative cervical myelopathy; mJOA, modified Japanese Orthopaedic Association.

### Sample size

A sample size of approximately 30 participants was identified as likely sufficient based on previous mixed-methods studies describing the development of patient decision aids.^24 25 38^ This was later confirmed by data saturation.

### Data collection

We collected quantitative and qualitative data. The data collection process involved pre-interview questionnaires (onlinesupplemental files 2  3), semi-structured interviews (onlinesupplemental files 4  5) and acceptability questionnaires (onlinesupplemental files 6  7) for patient-participants and health professional-participants. The primary quantitative outcome was the overall acceptability of the patient decision aid as assessed by rating the content, length, amount and balance of information, potential use of decision aid, impact on decision difficulty and implementation (table 4 and figure 2).

#### Pre-interview questionnaires

Pre-interview questionnaires were completed electronically by potential participants to gather baseline information. Collected data on demographics (eg, age, gender, education), clinical information (eg, had surgery for DCM) and professional background (tables 2 and 3) were used to purposively sample participants where possible.

#### Semi-structured interviews

Semi-structured interviews were conducted in accordance with IPDAS guidance^39 40^ to gather feedback on the design and content of the decision aid. A pilot interview was conducted to standardise the interview style between interviewers. All interviews were conducted online via video conference (Zoom)—minus one conducted in-person—by a male (ARG) or female (SM) physiotherapist researcher experienced in qualitative interviewing. Interviewers used interview guides and took notes to highlight key concepts or direct further questioning.

All participants provided written and verbal consent for interviews to be audio recorded and transcribed verbatim for analysis. A draft decision aid was provided to participants prior to their interview. A ‘think out loud’ method was used to gain feedback from participants as they viewed each page of the decision aid. New versions of the decision aid were created following interviews and were contrasted to previous versions in subsequent interviews (online supplemental file 8). Interviews lasted between 18 and 59 min. All participants had the opportunity to review their interview transcript prior to data analysis. No financial incentive was provided for participation.

#### Acceptability questionnaires

Following each interview, an acceptability questionnaire was provided electronically to participants during or after the interview. The acceptability questionnaires were adapted from The Ottawa Hospital Research Institute.^41^ Patient-participants rated sections of the decision aid (‘poor’, ‘fair’, ‘good’ or ‘excellent’), the length of the decision aid, and balance and usefulness of the information presented. Health professional-participants used a five-point Likert scale (strongly agree=5; strongly disagree=1) to assess agreement with various statements.

### Data analysis

Pre-interview and acceptability questionnaire responses were summarised using descriptive statistics including medians, IQRs, counts and percentages. Due to a low sample size and data that were not normally distributed, we reported median (IQR) and did not calculate CIs. No inferential statistics were reported as our analysis of quantitative data was purely descriptive.

**Figure 1:**
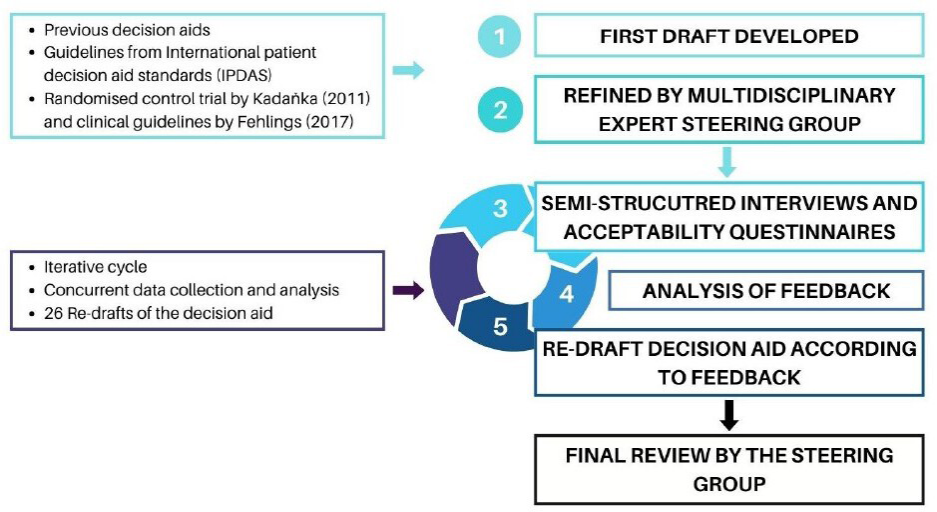
Process of the formation of subthemes and themes from participant interviews.

All qualitative aspects of this study were reported according to the 32-item Consolidated Criteria for Reporting Qualitative Research (COREQ) checklist (online supplemental file 9).^42^ All interview data were analysed using thematic analysis; a method for identifying, analysing and reporting patterns within data.^43^ The positionality of the interviewer and authorship team aligned with the best available evidence highlighting that people with mild DCM may not need surgery and should be engaged in shared decision-making, and people with moderate or severe DCM require timely surgery.^17 19^ Two physiotherapist researchers (AG and SM) independently recorded initial themes, familiarised themselves with the interviews via audio recordings or transcripts and developed a framework to organise concepts into broader themes and subthemes in Microsoft Excel. No qualitative software was used in addition to Excel due to the pragmatic and simplistic nature of our analysis (ie, analysing feedback on the decision aid). The mapping of themes and subthemes (figure 1) was achieved using a descriptive approach and multiple iterative cycles as new data emerged. Interview themes were not interpreted or validated by participants to protect the legitimacy of the authorship’s team interpretation as a central analytic process and allow themes to reflect patterns across the dataset rather than individual preferences. Any disagreements were discussed and resolved with a third author (JZ). The decision aid was continually updated before new interviews and circulated to the study authors to reach consensus. Consensus was determined by the majority of the study authors agreeing with proposed changes. No further interviews were conducted once data saturation was achieved (ie, no new feedback emerged that would lead to a new theme or subtheme from three consecutive participants) and participants had an overall positive impression of the decision aid. Saturation was assessed separately for patient-participants and health professional-participants. Triangulation^44^ was used to inform the interpretation of results by considering whether the quantitative results were consistent with the overall impression of the decision aid by participants during interviews.

### Patient and public involvement

Patient-participants and health professional-participants provided feedback on the decision aid throughout development and provided feedback on the manuscript.

## Results

### Adherence to the IPDAS criteria and user-centredness

Our decision aid (online supplemental file 10) met all six criteria to be classified as a decision aid which reduces the risk of harmful bias. 21 of the 23 quality criteria of the IPDASi checklist (V.4.0)^45^ (online supplemental file 11) were met and the criteria not met involved evaluating the effectiveness of the decision aid (eg, in a clinical trial). Readability was assessed including all the decision aid text (Grade 11.8) and without necessary complex words (Grade 8.7) using the SHeLL Editor (https://shell.techlab.works). Our decision aid met 10 of the 11 criteria for user-centredness (online supplemental file 12) as assessed by the User-Centred Design 11-item measure.^46^

### Participant characteristics and decision aid acceptability

**Figure 2:**
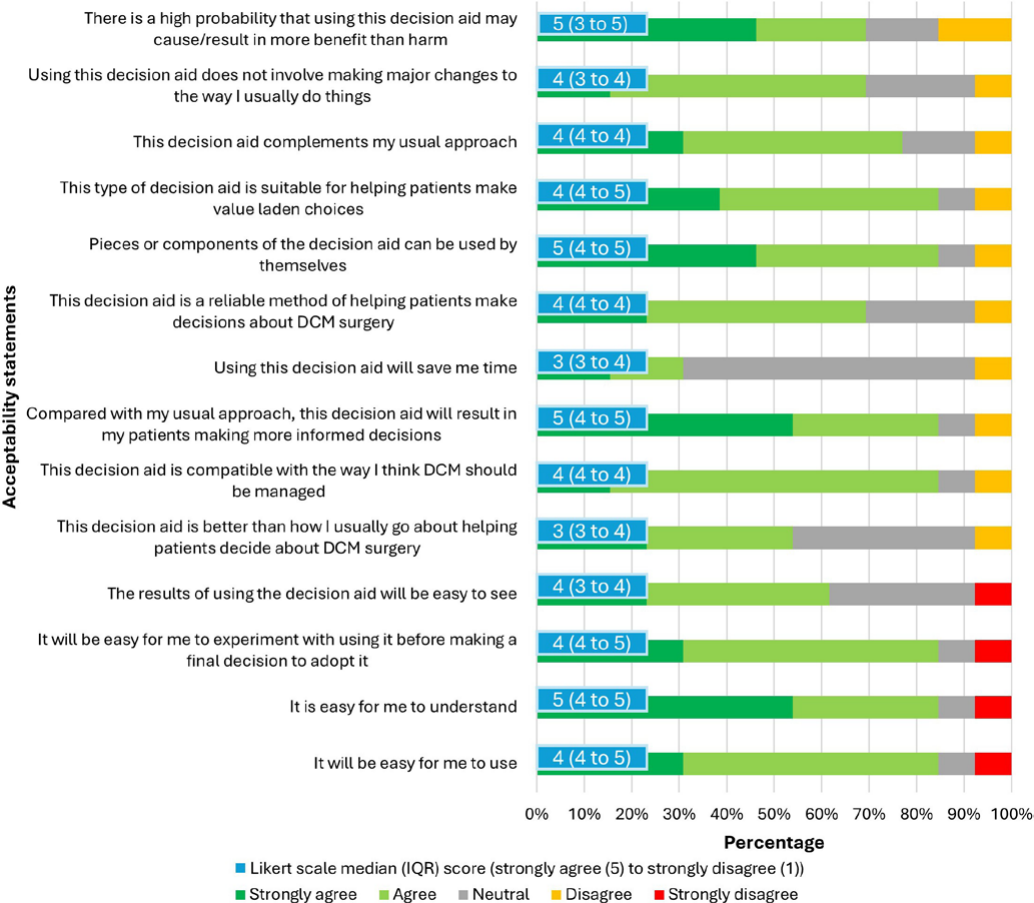
Acceptability questionnaire for health professional-participants that manage people with degenerative cervical myelopathy (n=11)^a^. ^a^Two health professional-participants did not complete the anonymous acceptability questionnaire. DCM, degenerative cervical myelopathy.

A total of 32 initial interviews were conducted; 19 patient-participants and 13 health professional-participants who manage people with DCM (tables2  3). Interview participants were selected from 54 people with DCM and 16 health professionals who completed the pre-interview survey. Two male participants attended an interview but withdrew before the interview began because we were unable to offer any compensation for their time. No participant was excluded. Interviews were conducted from March to September 2024. The results from the acceptability questionnaires are presented in table 4 and figure 2.

**Table 2:** Characteristics of patient-participants with degenerative cervical myelopathy

Characteristics All statistics are reported as n (%) unless specified otherwise	Patient-participants (n=19)
Age in years, mean (SD)	55 (10)
Female	17 (89%)
Country of residence	
United Kingdom	11 (58%)
Australia	2 (11%)
USA	2 (11%)
Canada	1 (5%)
Cyprus	1 (5%)
Ireland	1 (5%)
Sweden	1 (5%)
Highest level of education	
University graduate or postgraduate degree/s	11 (58%)
TAFE/Trade	4 (21%)
High school (completed)	4 (21%)
Employment status	
Employed full-time	3 (1%)
Employed part-time or casual	4 (21%)
Retired	5 (26%)
Sick/disability leave	2 (11%)
Other (eg, self-employed)	5 (26%)
Time from noticing symptoms until diagnosis of DCM	
<1 month	1 (5%)
1–3 months	3 (16%)
4–6 months	2 (11%)
7–12 months	2 (11%)
13–24 months	0 (0%)
>24 months	11 (58%)
Familiarity with mJOA scale	12 (63%)
Had surgery for DCM	15 (79%)
Satisfaction with treatment choice	
Extremely unsatisfied	5 (26%)
Somewhat unsatisfied	4 (21%)
Neither satisfied nor unsatisfied	5 (26%)
Somewhat satisfied	3 (16%)
Extremely satisfied	2 (11%)

DCM, degenerative cervical myelopathy; mJOA, modified Japanese Orthopaedic Association; N, number of participants; SD, Standard Deviation; TAFE, Technical and Further Education; USA, United States of America.

**Table 3:** Characteristics of health professional-participants who manage people with degenerative cervical myelopathy

Characteristics	Health professional-participants (n=13)
Age in years, mean (SD)	49 (12)
Female	2 (15%)
Country of health professional training*	
Australia	4 (27%)
UK	3 (20%)
Canada	2 (13%)
USA	2 (13%)
Germany	1 (7%)
India	1 (7%)
New Zealand	1 (7%)
Switzerland	1 (7%)
Professional background	
Neurosurgeon	4 (31%)
Neurologist	2 (15%)
Physiotherapist	2 (15%)
Orthopaedic surgeon	1 (8%)
General practitioner	1 (8%)
Rehabilitation and pain specialist†	1 (8%)
Consultant occupational physician‡	1 (8%)
Chiropractor/researcher	1 (8%)
Years of experience, mean (SD)	18 (11)
Work setting	
Private practice	4 (31%)
Private hospital	0 (0%)
Public hospital	7 (54%)
Other	2 (15%)
Average number of people with DCM managed per year	
5–10	3 (23%)
11–20	1 (8%)
21–30	3 (23%)
31–40	1 (8%)
>40	5 (38%)
Median (IQR) percentage of people diagnosed with DCM they would advise to have surgery	60% (40% to 70%)
Median (IQR) percentage of people diagnosed with mild DCM they would advise to have surgery	20% (10% to 45%)

* All health professional-participants were practising in their country of training at the time of interviews. One health professional-participant practising in Australia also trained in India and the UK.

† Rehabilitation and pain specialists, specialises in spinal injuries, regional pain and the management of acute and persistent pain.

‡ Consultant occupational physicians, specialises in the management and rehabilitation of injured workers and general management of spine conditions.

DCM, degenerative cervical myelopathy; N, number of participants.

**Table 4:** Acceptability questionnaire for patient-participants with degenerative cervical myelopathy (n=18)

Acceptability questionnaire items	N (%)
Section of decision aid rated as ‘excellent’ or ‘good’	
Degenerative cervical myelopathy: should I have surgery?	15 (83%)
What is DCM? How is DCM diagnosed?	16 (89%)
What are the categories of DCM?	16 (89%)
Which DCM category are you in when using the mJOA tool?	11 (61%)
What is recommended?	12 (67%)
Description of non-surgical management and DCM surgery	15 (75%)
Potential harms and benefits of non-surgical management and DCM surgery	12 (67%)
Questions for when you talk with a health professional	5 (83%)*
The length of the decision aid was just right	16 (89%)
The amount of information was just right	15 (83%)
I found the decision aid	
Balanced	13 (72%)
Slanted towards surgery	3 (17%)
Slanted towards the non-surgical option	2 (11%)
Would have found the decision aid ‘extremely’ or ‘very’ useful when making the decision about DCM surgery	12 (67%)
Agreed that the decision aid would have made their decision easier	16 (89%)
Enough information is provided to help people with DCM decide on whether to have surgery	13 (72%)

One patient-participant did not complete the anonymous acceptability questionnaire.

* Only six patient-participants completed this question as it was added later in the development process.

DCM, degenerative cervical myelopathy; mJOA, modified Japanese Orthopaedic Association; N, number of patient-participants.

### Feedback for each section of the decision aid

**Figure 3:**
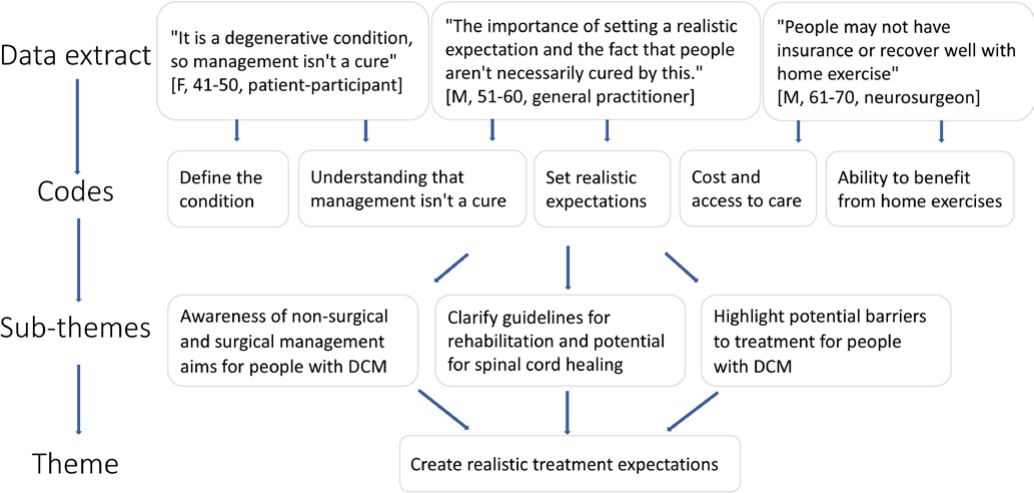
Formation of subthemes and themes. DCM, degenerative cervical myelopathy; F, female; M, male.

Most suggestions from participants were incorporated into the decision aid but some were not possible to implement as they conflicted with the best available evidence or other participants’ feedback. Patient-participant and health professional-participant feedback was analysed separately but presented in a combined summary below. Online supplemental file 13 outlines instances where patient-participant and health professional-participant feedback did or did not align, why some feedback was not incorporated into the decision aid and our justification for this. For example, patient-participants and health professional-participants agreed access and affordability to healthcare are barriers to optimal treatment but had different views on how the mJOA tool should be presented in the decision aid. Patient-participants did not think it was necessary to feature the mJOA prominently while health professional-participants thought the opposite. Figure 3 provides an example of how coding was used to develop themes and subthemes.

### Thematic analysis of interviews

Six themes were developed from interviews (online supplemental file 14).

#### Themes 1: Positive feedback

Most participants gave positive feedback on the content and design of the decision aid.

“I like the graphics that you’ve got on there. And I like the fact that it’s putting the decision-making back with the patient but giving them information.” (F, 51–60, patient-participant)

“If neurologists had access to data like this…we would be better suited to help them or help triage them appropriately.” (F, 31–40, neurologist)

#### Theme 2: Modify the decision aid to enhance understanding for people with DCM

All participants suggested modifying wording to make the decision aid more patient friendly. Patient-participants and health professional-participants suggested using more practical examples (eg, difficulty buttoning up a shirt), considering colours appropriate for people with colour blindness and modifying some graphics. One health professional-participant suggested the decision aid could be presented in languages other than English. All participants identified potential changes and most provided suggestions to simplify the decision aid.

“I did wonder like whether a QR code would be helpful, that could take them through to either a website or an option for larger texts or different languages.” (F, 41–50, physiotherapist)

“Be careful with language - is it a slowly progressing spinal cord injury (not a) scary slow motion spinal cord injury” (M, 21–30, physiotherapist)

“I think it could be simplified, maybe more visual.” (F, 51–60, patient-participant)

“The document itself is dense with a lot of material but there are ways to simplify it.” (M, 61–70, neurosurgeon)

#### Theme 3: Improving awareness of DCM and acceptability of the decision aid

Patient-participants frequently suggested their diagnosis was delayed. Health professional-participants suggested some primary practitioners may not be aware of DCM, clarify who the decision aid is targeting and had mixed views on the most appropriate format. Most participants agreed that the wording used is appropriate to encourage timely action without people with DCM feeling panic, but some had conflicting views on using the mJOA or how it is incorporated.

“Awareness is an issue in primary care.” (M, 21–30, physiotherapist)

“I didn’t know what it was until I found out I had it, and I even have trouble explaining it to friends now, so I just say I have a spinal cord injury.” (F, 41–50, patient-participant)

“I think what potentially we should have, is a better explanation of the mJOA. And also explain how it’s determined.” (F, 51–60, patient-participant)

#### Theme 4: Highlight variations in symptoms and promote individual management

All patient-participants highlighted the impact of their symptoms which were variable. Primary care health professional-participants highlighted the difficulties of early diagnosis of DCM due to the diversity of symptoms. Most health professional-participants agreed management should involve shared decision making but some surgeons suggested aspects of care are more appropriate for a specialist to decide (eg, the surgical approach). Some health professional-participants suggested including quality of life measures or other surveys to guide treatment alongside the mJOA.

“The clumsiness and tripping over were the main symptoms for me.” (F, 51–60, patient-participant)

“This should be an individualised discussion specific to that patient…thresholds can be subjective, and people need to get the right information.” (M, 21–30, physiotherapist)

“I talk to them about symptoms worsening or the impact of their life, but the score is just where they stand.” (M, 41–50, neurosurgeon)

#### Theme 5: Create realistic treatment expectations

Patient-participants and health professional-participants frequently highlighted that DCM is a progressive condition needing tailored management. They also agreed with descriptions of non-surgical management and suggested including perioperative recommendations. Some patient-participants mentioned they were unaware of rehabilitation requirements while others felt post-surgical care was beneficial. Some health professional-participants had different views on how to explain surgery, but most suggested surgery aims to slow DCM progression. Some health professional-participants suggested focusing on acute healing times while patient-participants highlighted the time to adapt to life post-surgery.

“The importance of setting a realistic expectation and the fact that people aren’t necessarily cured by this.” (M, 51–60, general practitioner)

“Post op care and rehab makes a huge difference – persistence with exercises and recommendations from OT.” (F, 41–50, patient-participant)

“Rehabilitation is important post-surgery, but type and timing should be personalised. People may not have insurance or recover well with home exercise.” (M, 61–70, neurosurgeon)

#### Theme 6: Facilitate equitable access to care and active management strategies

Patient-participants and health professional-participants suggested timely access to care varies between public and private healthcare settings and countries. Both suggested incorporating prompts to monitor symptoms with a health professional within the decision aid (eg, note key changes). Health professional-participants suggested monitoring at 6-month intervals and considering individual circumstances. Patient-participants frequently suggested that social groups are helpful.

“So, in the NHS, no rehabilitation is provided, nothing. Yeah. After my first operation, I was so traumatised… I was so traumatised after surgery. And adapting can take time.” (F, 71–80, patient-participant)

“They may not need to see anyone, but they need to know the risk factors and when to come back.” (M, 41–50, neurosurgeon)

## Discussion

### Summary of findings

Patient-participants and health professional-participants involved in the management of DCM highlighted a need to increase awareness of the condition, early diagnosis and effective monitoring. Six themes were developed from interviews (figure 1). Themes covered feedback on the decision aid, symptom diversity, outcome expectations and active management participation. Most patient-participants rated the decision aid as good-to-excellent and 89% said it would have made their management decision easier. Most health professional-participants strongly agreed or agreed the decision aid is acceptable and suggested the aid could better align treatment expectations with evidence.

### Meaning of the study

This is the first patient decision aid for people with DCM. It is designed to facilitate shared decision making, particularly for those with mild DCM where it is unclear if surgical or non-surgical management is best. It includes prompts for monitoring DCM with health professionals and consideration for its implementation. It was suggested that the decision aid could increase awareness of DCM which is prioritised in the literature.^13^

Most patient-participants (58%) received their diagnosis over 24 months after noticing symptoms and only one patient-participant (5%) was diagnosed within 1 month. Interviews suggested presenting the decision aid in different media (eg, video) and distributing it electronically via multi-disciplinary teams (ie, health professionals and non-clinical staff such as administrative staff) may increase engagement. The decision aid will be available online via the Institute for Musculoskeletal Health webpage and Myelopathy.org. Myelopathy.org is the first global charity dedicated to improving the quality of life of people with DCM and was recognised in a Lancet Neurology editorial as pivotal to driving future change in improving outcomes for people with DCM.^47^

Future acceptability of our decision aid may be facilitated by enhancing visual appeal, simplicity of design and ease of use. For example, 89% of patient-participants who completed the acceptability questionnaire said our decision aid would have made their decision easier (table 4) and all health professional-participants ‘strongly agreed’ or ‘agreed’ it would be easy to use and understand. Actioning feedback to facilitate shared decision-making and reduce complexity (eg, an external link to the mJOA) may mean the aid is more feasible to be integrated into routine care than other more complex educational tools.^22^ Incorporating plain language, bullet points and appropriate visuals could also facilitate acceptability.^48^ Dot point questions about quality of life and pain included taking into account the ‘ceiling-effect’ of the mJOA for people with mild DCM.^49^

Health professional-participants frequently suggested the decision aid should only focus on mild DCM but patient-participants advocated that people with moderate DCM could also be managed without surgery in some instances. To address this discussion point, the decision aid includes the only known RCT that had compared surgical and non-surgical management for people with mild-moderate DCM. This RCT reported no significant between-group differences in outcomes at 10 years.^19^ To separate mild and moderate DCM, we used the most recent clinical guidelines that recommend people with moderate DCM undergo surgery. The majority of the authorship group agreed the decision aid is most useful to help people with mild DCM decide if they need surgery or non-surgical management plus monitoring.^50^ A recent systematic review and meta-analysis presents estimates of people with mild DCM who experience neurological decline without surgery (9% at 1 year, 16% at 2 years, 21% at 3 years, 27% at 5 years, 36% at 15 years and 37% at 20 years).^34^ Encouraging appropriate screening could further encourage earlier detection of DCM and facilitate appropriate management.^51^

Interviews frequently highlighted non-surgical management and perioperative rehabilitation for people with DCM is necessary but inconsistently provided. Limited research guides the type of rehabilitation patients should receive.^52^ A recent retrospective cohort study (n=116) demonstrated that people with DCM participating in a comprehensive rehabilitation can achieve considerable functional improvement following spinal surgery.^52^ Patient-participants reported benefits from several allied health services like physiotherapy or occupational therapy (“Through ongoing activity, particularly focused on addressing the symptoms of myelopathy, I’ve been able to behave in pretty much a normal way. I’m active, I run again.” - F, 61–70, patient-participant). However, early management is important as people with mild DCM managed without surgery (n=167) are more likely to report benefits from physiotherapy than those with moderate or severe DCM.^4^ People with moderate or severe DCM should consult a surgeon but may benefit from support from non-surgical clinicians before, after or in the absence of available surgical management options.^52^

Affordable care is a barrier to optimal management but facilitating social connection could improve quality of life for people with DCM. Affordability and waiting times for surgery in the public sector can be substantial in many countries. People with DCM of various severity can become increasingly anxious as they wait for a diagnosis. Health professional-participants suggested strategies to empower people with DCM to manage and monitor their own symptoms (eg, recording symptom changes). The decision aid includes numeric estimates of benefits and harms, links to social groups, and a statement that research on neural plasticity and rehabilitation is ongoing. Including these features may reduce potential anxiety and improve quality of life (eg, mood and motivation) for people living with DCM irrespective of their situation.^52^

Our patient decision aid for people with DCM considering surgery could be a useful resource to promote continuity of care for people with DCM and align treatment with evidence-based guidelines. Patient decision aids have been shown to lead to large increases in patient knowledge, expectations of benefits and harms, and participation in making the decision.^21^ Multiprofessional use of patient decision aids, including this DCM decision aid, can promote ‘shared understanding’ of decisions and introduce the concept of choice at the beginning and during consultations with various health professionals during a patients’ journey.^22^ Participant interviews supported this, suggesting that multiprofessional treatment is key for people with DCM and our patient decision aid is feasible to facilitate discussions with a variety of health professionals (eg, general practitioners, surgeons, allied health clinicians).

### Strengths and limitations

Our development process has several strengths. We developed a decision aid that satisfies the IPDAS criteria, used mixed methods to evaluate acceptability of the decision aid, used data saturation to guide the number of interviews conducted and used triangulation^44^ to improve the rigour of the coding process. The study authors include a multidisciplinary team of health professionals and people with lived experience of DCM. Patient-participants involved in the development process were from diverse backgrounds across the world and provided feedback at multiple stages (eg, interviews, review of the final manuscript and decision aid). One-on-one interviews conducted with participants also allowed for in-depth feedback. The readability of the decision aid was measured and improved during development. The decision aid includes key elements which aim to address low health literacy such as subheadings, bullet points and white space for ease of use.^48^ We also provide justification of the evidence used to inform the benefits and harms presented.

Limitations include challenges with recruitment. For example, despite using purposive sampling, most patient-participants were females (89%), most health professional-participants were males (85%) and most patient-participants had undergone surgery (79%). Health professional-participants were from eight different professions and eight different countries of training which has advantages and disadvantages. Recruiting from diverse settings increases the generalisability of our study results for potential use of the tool with different health professionals in several countries but reduces region-specificity and may limit acceptability to low-to-middle income countries. Nonetheless, we prioritised developing an acceptable decision aid for use in different countries to increase reach, promote earlier diagnosis and encourage evidence-based management for people with DCM globally. Future research should include the occupation of patient-participants as this appears to be an important risk factor for DCM development based on previous research.^53^ The decision aid and other future resources will need to be translated to other languages for people from non-English speaking backgrounds. The decision aid appears to be acceptable but there is still uncertainty in the existing evidence comparing surgery to non-surgical management and a need to consider individualised management.

## Conclusion

Our decision aid for people with DCM was rated as an acceptable tool by patient-participants and health professional-participants involved in their management. This decision aid is feasible for use by clinicians and people diagnosed with DCM to help with shared decision making. The tool is likely acceptable and feasible for people with mild DCM, given surgical management may not yet be mandatory, and a variety of non-surgical management options are still available. During the study, our interviews reinforced the need to increase awareness of DCM, improve monitoring of symptoms and ensure ease of access to the decision aid to optimise global impact. We encourage the use of the decision aid in daily clinical practice, while a larger study evaluating the potential benefits of the decision aid is being planned.

## Supplementary material

10.1136/bmjopen-2025-106337 online supplemental file 1

10.1136/bmjopen-2025-106337 online supplemental file 2

10.1136/bmjopen-2025-106337 online supplemental file 3

10.1136/bmjopen-2025-106337 online supplemental file 4

10.1136/bmjopen-2025-106337 online supplemental file 5

10.1136/bmjopen-2025-106337 online supplemental file 6

10.1136/bmjopen-2025-106337 online supplemental file 7

10.1136/bmjopen-2025-106337 online supplemental file 8

10.1136/bmjopen-2025-106337 online supplemental file 9

10.1136/bmjopen-2025-106337 online supplemental file 10

10.1136/bmjopen-2025-106337 online supplemental file 11

10.1136/bmjopen-2025-106337 online supplemental file 12

10.1136/bmjopen-2025-106337 online supplemental file 13

10.1136/bmjopen-2025-106337 online supplemental file 14

## Data Availability

Data are available upon reasonable request.
