# Supplementary material for: Development and acceptability of a patient decision aid for people with degenerative cervical myelopathy: an international mixed-methods study
Source: BMJ Open. 2026 Apr 3;16(4):e106337. doi: 10.1136/bmjopen-2025-106337 (PMC13052582; doi:10.1136/bmjopen-2025-106337)
Supplement: online supplemental file 1 [file bmjopen-16-4-s001.docx]

Supplementary file 1: Literature search strategy

Search for: 8 and 13 and 18

Results: 27

| Ovid MEDLINE(R) ALL <1946 to December 09, 2024> | | | |
| --- | --- | --- | --- |
| **#** | **Search Statement** | **Results** | **Annotation** |
| 1 | clinical trial.pt. | 540827 |  |
| 2 | randomised.ab,ti. | 143412 |  |
| 3 | randomly.ab,ti. | 449326 |  |
| 4 | trial.ab,ti. | 841770 |  |
| 5 | humans/ | 22410505 |  |
| 6 | 1 or 2 or 3 or 4 or 5 | 22742159 |  |
| 7 | animals/ | 7563615 |  |
| 8 | 6 not 7 | 20310887 |  |
| 9 | exp spinal cord compression/ | 12077 |  |
| 10 | myelopathy.mp. | 17318 |  |
| 11 | radiculomyelopathy.mp. | 160 |  |
| 12 | myeloradiculopathy.mp. | 377 |  |
| 13 | or/9-12 | 27326 |  |
| 14 | exp surgery/ | 41215 |  |
| 15 | exp surgical procedures,operative/ | 3682311 |  |
| 16 | surgery.mp. | 3240347 |  |
| 17 | surgical.mp. | 1685832 |  |
| 18 | or/14-17 | 5303979 |  |
| 19 | 8 and 13 and 18 | 12209 |  |
