# Supplementary material for: Development and acceptability of a patient decision aid for people with degenerative cervical myelopathy: an international mixed-methods study
Source: BMJ Open. 2026 Apr 3;16(4):e106337. doi: 10.1136/bmjopen-2025-106337 (PMC13052582; doi:10.1136/bmjopen-2025-106337)
Supplement: online supplemental file 2 [file bmjopen-16-4-s002.docx]

Supplementary file 2: People with DCM pre-interview questionnaire

**Consent section**

1. Please make sure you have read the Patient Participant information statement before starting the survey.
2. PATIENT PARTICIPANT CONSENT FORM

**PARTICIPANT CONSENT FORM**

**Degenerative Cervical Myelopathy: what information is required to make an informed management decision?**

In giving my consent, I confirm that that have read and understood the Participant Information Sheet on the abovenamed research study and understand I can discuss the study with researchers of the study using contact details provided in the Participant Information Sheet if I have any questions about the study.

- I have been made aware of the procedures involved in the study, including any known or expected inconvenience, risk, discomfort, or potential side effect and of their implications as far as they are currently known by the researchers.
- I understand that the interview/focus group discussion will be audio-recorded and video-recorded, will then be transcribed (e.g. using Otter AI – a transcription software) and be kept in a manner in which I cannot be identified for analysis, and I agree to this.
- I understand that the University of Sydney software license for Qualtrics will be used to manage the collection and storage of my research data.
- I have had an opportunity to ask questions and I am satisfied with the answers I have received.
- I freely choose to participate in this study and understand that I can withdraw at any time.
- I also understand that the research study is strictly confidential.
- I consent to the storage and use of my information collected from me for use, as described in the relevant section of the Participant Information Sheet, for:

- This specific research project

- Other research that is closely related to this research project

- Any future research

I hereby agree to participate in this research study.

- Yes, I would be happy to participate in this study
- No, I would prefer not to participate in this study

1. I would like to review my interview or focus group transcripts

- Yes
- No

1. I consent to being contacted for future studies

- Yes
- No

1. I consent to the future use of any data by the researchers or other collaborators to use the data I provide for research purposes. I understand that before the investigators or their collaborators use any data that I provide, they must seek additional ethics approval.

- Yes
- No

1. I am willing to participate in a focus group (up to 2hrs in duration) instead of an interview (approximately 30-minutes in duration):

- Yes
- No

1. I would like to be emailed a copy of the study results when they become available:

- Yes
- No

1. I would like to be:

- Acknowledged in the publication (participate in an interview)
- An author (participate in an interview and contribute to further work on the publication)

**Pre-interview Questionnaire**

**Study ID: _______________**

Thank you for your participation in this study, which is investigating what information is important for people with DCM when considering surgery.

We would like you to answer a few questions before the interview. This should not take more than 5-minutes.

**First, when are the best times to schedule you for an online interview…**

*Please provide below your best contact details for a researcher from the University of Sydney to contact you and arrange the follow-up interview:*

Name: _____________________________________

Email: _____________________________________

Best contact telephone number: _________________________________

Best time/s to call: _________________________________

Please mark the times that are suitable to arrange an interview in the boxes below:

|  | **Monday** | **Tuesday** | **Wednesday** | **Thursday** | **Friday** |
| --- | --- | --- | --- | --- | --- |
| **8 – 10am** |  |  |  |  |  |
| **10 – 12pm** |  |  |  |  |  |
| **12 – 2pm** |  |  |  |  |  |
| **2 – 4pm** |  |  |  |  |  |
| **4 – 6pm** |  |  |  |  |  |

**Please answer some quick questions about you...**

1. Please indicate your gender:

- Female
- Male
- Non-binary

1. Please indicate your age: [free text response]

___________________

1. In which country do you currently live? [free text response]

___________________

1. What option best describes your highest level of education?

- Primary school or less
- High school (not completed)
- High school (completed)
- TAFE/Trade
- University- undergraduate degree/s (completed)
- University- postgraduate degree/s e.g. Masters, PhD (completed)
- Other (please specify) ____________________________

1. What is your employment status?

- Employed part-time
- Employed full-time
- Casual work
- Retired
- Unemployed
- Student
- Sick/disability leave
- Other (please specify) ____________________________

1. Do you have private health insurance?

- Yes
- No

1. How long ago were you diagnosed with DCM? __________________
2. How long before your diagnosis of DCM did you notice your symptoms?

- <1 month ago
- 1-3 months ago
- 4-6 months ago
- 6-12 months ago
- 12-24 months ago
- >24 months ago

1. Have you heard of the modified Japanese Orthopaedic Association scale that is used to rate the severity of DCM?
   - - - Yes
       - No
2. Have you had surgery for DCM?
   - - - Yes
       - No
3. How happy are you that your symptoms are currently being managed?

- Extremely unhappy
- Somewhat unhappy
- Neither happy or unhappy
- Somewhat happy
- Extremely happy

____________________________________________________________________________

**Thank you for completing the questionnaire.**
