# Supplementary material for: Development and acceptability of a patient decision aid for people with degenerative cervical myelopathy: an international mixed-methods study
Source: BMJ Open. 2026 Apr 3;16(4):e106337. doi: 10.1136/bmjopen-2025-106337 (PMC13052582; doi:10.1136/bmjopen-2025-106337)
Supplement: online supplemental file 3 [file bmjopen-16-4-s003.docx]

Supplementary file 3: Health professional pre-interview questionnaire

**Consent section**

1. Please make sure you have read the Health Professional Participant information statement before starting the survey.
2. HEALTH PROFESSIONAL PARTICIPANT CONSENT FORM

**PARTICIPANT CONSENT FORM**

**Degenerative Cervical Myelopathy: what information is required to make an informed management decision?**

In giving my consent, I confirm that that:

Tick/initial boxes

- The details of any involvement have been explained to me, and I have been provided with a written Participant Information Statement to keep.
- I understand the purpose of the study is to investigate what information is important for people with DCM who are considering surgery.
- I acknowledge that the risks and benefits of participating in this study have been explained to me to my satisfaction.
- I understand that in this study I will be required to answer a pre-interview questionnaire (5-minutes) and attend an interview to provide feedback on an educational pamphlet on treatment options for people with DCM (online or via telephone) that will last 30-minutes.
- I understand that my participation will involve my interview to be recorded.
- I understand that information may be used in future research and the data collected for this study may use it in future projects. By providing consent I allow my information to be shared locally and internationally with other research collaborators as needed. I understand that it is unknown at this stage what these other projects will involve, and ethical approval will be gained before my information in used in these future projects.
- I understand that being in this study is completely voluntary.
- I am assured that my decision to participate will not have an impact on any relationship with the research team or the University of Sydney or the Local Health District.
- I understand that I am free to withdraw from this study at any time and can choose to withdraw any information already provided (unless the data has already been de-identified or published).
- I have been informed that the confidentiality of the information provided by myself will be protected and will only be used for purposes that has been agreed to. I understand that information will only be told to others with my permission, except as required by law.
- I understand that the results of this study may be published, and that publications will not contain any identifiable information about me.

I hereby agree to participate in this research study.

- Yes, I would be happy to participate in this study
- No, I would prefer not to participate in this study

1. I would like to review my interview or focus group transcripts

- Yes
- No

1. I consent to being contacted for future studies

- Yes
- No

1. I consent to the future use of any data I provide for research purposes. I understand that before the investigators or their collaborators use any data that I provide, they must seek additional ethics approval.

- Yes
- No

1. I am willing to participate in a focus group (up to 2hrs in duration) instead of an interview (approximately 30-minutes in duration):

- Yes
- No

1. I would like to be emailed a copy of the study results when they become available:

- Yes
- No

1. I would like to be:

- Acknowledged in the publication (participate in an interview)
- An author (participate in an interview and contribute to further work on the publication)

**Pre-interview Questionnaire**

**Study ID: _______________**

Thank you for your participation in this study, which is investigating what information is important for people with DCM when considering surgery.

We would like you to answer a few questions before the interview. This should not take more than 5-minutes.

**First, when are the best times to schedule you for an online interview…**

*Please provide below your best contact details for a researcher from the University of Sydney to contact you and arrange the follow-up interview:*

Name: _____________________________________

Email: _____________________________________

Best contact telephone number: _________________________________

Best time/s to call: _________________________________

Please mark the times that are suitable to arrange an interview in the boxes below:

|  | **Monday** | **Tuesday** | **Wednesday** | **Thursday** | **Friday** |
| --- | --- | --- | --- | --- | --- |
| **8 – 10am** |  |  |  |  |  |
| **10 – 12pm** |  |  |  |  |  |
| **12 – 2pm** |  |  |  |  |  |
| **2 – 4pm** |  |  |  |  |  |
| **4 – 6pm** |  |  |  |  |  |

**Please answer some quick questions about you...**

1. Please indicate your gender:

- Female
- Male
- Non-binary

1. Please indicate your age: [free text response]

___________________

1. In which country did you receive your health professional training/qualification? [free text response]

___________________

1. What type of health professional are you?

- Neurosurgeon
- Orthopaedic surgeon
- Neurologist
- Rheumatologist
- General practitioner
- Physiotherapist
- Other (please specify) ____________________________

1. How many years have you been practicing? [free text response]

______________________

1. Which clinical setting have you spent the most time practicing in?

- Private practice
- Public hospital
- Private hospital
- Other (please specify) ____________________________

1. On average, how many patients with suspected or confirmed DCM do you manage/review per year? [free text response]

______________________

1. On average, of the people with diagnosed (mild, moderate or severe) DCM that you see – what percentage would you advise to have surgery?

Percentage (%): 0__10__20__30__40__50__60__70__80__90__100

1. If only seeing people with mild DCM – on average, what percentage would you advise to have surgery?

Percentage (%): 0__10__20__30__40__50__60__70__80__90__100

**Thank you for completing the questionnaire.**
