# Supplementary material for: Development and acceptability of a patient decision aid for people with degenerative cervical myelopathy: an international mixed-methods study
Source: BMJ Open. 2026 Apr 3;16(4):e106337. doi: 10.1136/bmjopen-2025-106337 (PMC13052582; doi:10.1136/bmjopen-2025-106337)
Supplement: online supplemental file 5 [file bmjopen-16-4-s005.docx]

Supplementary file 5: Health professional interview guide

**Example structure of interviews and focus groups with health professional participants**

*Note: The topics below will serve as an outline to guide interviews and focus groups*

***I. INTRODUCTION***

*Hello, my name is [name of interviewer]. Introduce self and others in the room if appropriate (e.g. other team members).*

1. *Introduce the project*
2. *The purpose of this study is to better understand what information is important for people with degenerative cervical myelopathy (DCM) when deciding to have surgery or use a non-surgical approach. Is this an appropriate time to interview you?*

*Yes CONTINUE*

*No SET UP A TIME LATER*

1. *Explain the purpose of the interview or focus group*
2. *The study aims to explore patient and health professional views on how treatment information should be presented.*
3. *Describe the audio recording and video recording, how we will assure confidentiality and answer any questions*
4. *This interview will be approximately 30 minutes (or 2 hours for focus groups), be audio recorded and transcribed (*e.g. using Otter AI – a transcription software) *so that we have an accurate record of your response. Please be assured that the recording and your transcript will be kept confidential. E.g., “Only researchers involved in the study will have access to your responses. Our study does have ethics approval. Once your interview has been transcribed, only a site identifier will be linked to the transcripts, while any information linking you to the transcript will be destroyed. The audio and video recording will be destroyed as soon as the transcript is verified and analyzed by research staff”.*
5. *If you need to contact me at any time after the interview or focus group to ask any questions, you can contact me via email (provide researcher email E.g., Mr. Andrew Gamble agam165@uni.sydney.edu.au)*
6. *You will be given the opportunity to review the recording/transcript/your responses prior to publication and analysis if you choose to do so.*
7. *If, at any time, you feel that the questions are too sensitive, I would be happy to turn off the recorder during that portion of questioning. You may also skip any questions you wish during the interview or focus group.

   Do you have any questions for me? [Answer any questions]*

**Opening questions**

- What is your understanding of the treatment options for people with DCM? What causes it? How can it be treated?
- What do you think of DCM surgery as a treatment?

**Brief explanation of DCM surgery to health professionals (depending on their current level of understanding e.g. do not explain this to a surgeon)**

“I am now going to give you a short explanation of DCM surgery and why it is indicated that has been standardised to read to each participant.”

“DCM surgery requires admission to hospital, anesthetic and small surgical cuts to the front or back of the neck to access the spinal cord. Immediately following surgery there can be pain, swelling, reduced movement and possible side effects such as difficulty swallowing. The aim of surgery is to take pressure off the spinal cord from structures that may be compressing it and causing symptoms.”

**Core questions**

If we were designing a decision aid to help patients decide whether to have DCM surgery or not….

- What information is most important for them to know? (prompt for views on presenting different treatment options, benefits and harms, recovery time, likelihood of need for revision surgery, details of the procedure, etc.)
- How would you like information to be presented in the decision aid in terms of visual aids, text, tables, pictures, etc.? (example below, but exact topics will depend on what arose from the previous question)
  - Different treatment options
  - Benefits and harms
  - Recovery time
  - Likelihood of need for revision surgery
  - Details of the procedure
- How would your response to the above options differ if the information was intended to be used during a consultation with a health professional?

**When reviewing the investigator-developed decision aid:**

Instructions to health professionals (as an example): The material we want you to review has been developed for patients to improve their knowledge and confidence in making the decision to have DCM. We would like for you to help us better understand your experience of this material – for example, how you find the visual appeal, readability, content, and what are your overall experiences using this material**.**

To do this, I am going to ask you to think out loud while you read through the material. Just say everything that goes through your mind- if you are finding anything challenging, what your eye is drawn to. If a page is easy, and you understand what to do – just say that. Providing examples is very helpful (e.g. “look at a table”, “look at a page with just text vs with an image”).

Prompt questions as health professionals are reading through the material:

- How do you think patients would find this section?
- Did you feel like patients will know where to look, and what to do next?
- Did you feel like patients knew the relevance of this section in their decision?
- How do you think patients will find the content of this section?
- Were the instructions clear/helpful?
- How easy was it to understand the section? (readability)
- Was there anything that was unclear or confusing?
- How were the visual aids? (Any content supplementing written material in the decision aid)
- How was the functionality?
- Is there anything that you would improve in this section?
- What did you like most about this material?
- What did you like least about this material?

**General feedback at the end**

- Are there any topics that you would like to see in future versions of this tool?
- Do you have any other general feedback, thoughts, or comments?
