# Supplementary material for: Development and acceptability of a patient decision aid for people with degenerative cervical myelopathy: an international mixed-methods study
Source: BMJ Open. 2026 Apr 3;16(4):e106337. doi: 10.1136/bmjopen-2025-106337 (PMC13052582; doi:10.1136/bmjopen-2025-106337)
Supplement: online supplemental file 6 [file bmjopen-16-4-s006.docx]

Supplementary file 6: Acceptability questionnaire for people with DCM

We would like to know what you think about the patient decision aid you have just read.

1. Please rate each section by circling ‘poor’, ‘fair’, ‘good’, or ‘excellent’ to show what you think about the way the information was presented on:

| Degenerative Cervical Myelopathy (DCM): Should I have surgery? | Poor | Fair | Good | Excellent |
| --- | --- | --- | --- | --- |
| What is DCM? The diagnosis and symptoms of DCM | Poor | Fair | Good | Excellent |
| What are the categories of DCM? | Poor | Fair | Good | Excellent |
| Which DCM category are you in? | Poor | Fair | Good | Excellent |
| What are the recommended management options? | Poor | Fair | Good | Excellent |
| What do the management options involve? | Poor | Fair | Good | Excellent |
| Comparing non-surgical management to DCM surgery | Poor | Fair | Good | Excellent |
| Questions to consider when talking with your health professional | Poor | Fair | Good | Excellent |

1. The length of the decision aid was (select one):
   1. Too long
   2. Too short
   3. Just right
2. The amount of information was (select one):
   1. Too much information
   2. Too little information
   3. Just right
3. I found the decision aid (select one):
   1. Slanted towards the non-surgical option
   2. Slanted towards surgery
   3. Balanced
4. How useful do you think this decision aid is (or would have been) when making a decision about DCM surgery?
   1. Not at all useful
   2. Slightly useful
   3. Moderately useful
   4. Very useful
   5. Extremely useful
5. Did this decision aid/would this decision aid make deciding whether to have surgery…?
   1. Easier
   2. More difficult
   3. Comments:
6. Do you think we provided enough information to help people with DCM decide on whether to have surgery or not?
   1. Yes
   2. No
   3. Comments:
