# Supplementary material for: Development and acceptability of a patient decision aid for people with degenerative cervical myelopathy: an international mixed-methods study
Source: BMJ Open. 2026 Apr 3;16(4):e106337. doi: 10.1136/bmjopen-2025-106337 (PMC13052582; doi:10.1136/bmjopen-2025-106337)
Supplement: online supplemental file 7 [file bmjopen-16-4-s007.docx]

Supplementary file 7 Acceptability questionnaire for health professionals

The following set of questions asks about your perceptions of the decision aid you just read. We are interested in your reactions to the decision aid. Please indicate how strongly you agree or disagree with each statement by *circling* the appropriate number.

| **In general:** | **Strongly disagree** |  | **🡪** |  | **Strongly agree** |
| --- | --- | --- | --- | --- | --- |
| It will be easy for me to use | 1 | 2 | 3 | 4 | 5 |
| It is easy for me to understand | 1 | 2 | 3 | 4 | 5 |
| It will be easy for me to experiment with using it before making a final decision to adopt it | 1 | 2 | 3 | 4 | 5 |
| The results of using the decision aid will be easy to see | 1 | 2 | 3 | 4 | 5 |
| This decision aid is better than how I usually go about helping patients decide about DCM surgery | 1 | 2 | 3 | 4 | 5 |
| This decision aid is compatible with the way I think DCM should be managed | 1 | 2 | 3 | 4 | 5 |
| Compared with my usual approach, this decision aid will result in my patients making more informed decisions | 1 | 2 | 3 | 4 | 5 |
| Using this decision aid will save me time | 1 | 2 | 3 | 4 | 5 |
| This decision aid is a reliable method of helping patients make decisions about DCM surgery | 1 | 2 | 3 | 4 | 5 |
| Pieces or components of the decision aid can be used by themselves | 1 | 2 | 3 | 4 | 5 |
| This type of decision aid is suitable for helping patients make value laden choices | 1 | 2 | 3 | 4 | 5 |
| This decision aid complements my usual approach | 1 | 2 | 3 | 4 | 5 |
| Using this decision aid does not involve making major changes to the way I usually do things | 1 | 2 | 3 | 4 | 5 |
| There is a high probability that using this decision aid may cause/result in more benefit than harm | 1 | 2 | 3 | 4 | 5 |

**Please provide any further comments or feedback in the comments section below:**

_________________________________________________________________________________________________________________________________________________________________________________________________________________________________
