# Supplementary material for: Development and acceptability of a patient decision aid for people with degenerative cervical myelopathy: an international mixed-methods study
Source: BMJ Open. 2026 Apr 3;16(4):e106337. doi: 10.1136/bmjopen-2025-106337 (PMC13052582; doi:10.1136/bmjopen-2025-106337)
Supplement: online supplemental file 8 [file bmjopen-16-4-s008.pdf]

Degenerative Cervical Myelopathy (DCM):
Do I need surgery now?

- This decision aid is for people with DCM considering surgery
- All information should be discussed with a health professional

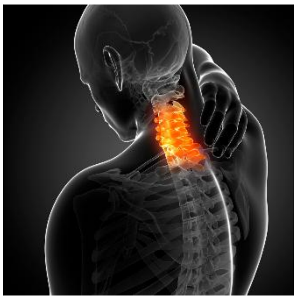

What is DCM?

- DCM results from changes in the spinal canal of the neck that compress the spinal cord.
- This 'slow motion spinal cord injury' can disturb nerve supply from the brain to the neck, shoulders, arms, hands or legs.

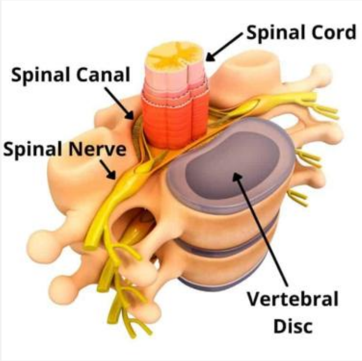

Diagnosis of DCM is based on:

- **Imaging**: MRI and other scans can show <u>compression of the spinal cord</u>.

  * **Caution -** image findings should <u>not determine treatment choice alone</u> as they may be due to normal aging or not directly causing your symptoms.

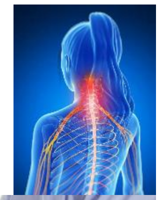

Compression of the spinal cord can lead to:

- **Symptoms:** pain or stiffness in the neck/shoulder/arms/hands or other areas, fatigue, pins and needles, numbness, reduced strength, clumsiness, difficulty using your hands or issues walking or toileting.

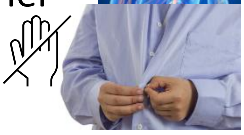

Questions to consider when talking with your health professional:

- What are the benefits and harms of surgery and non-surgical management?
- What do you offer as non-surgical management and how frequently will I be reassessed?
- What other factors do I need to consider? (age, weight, demands of leisure activity and work, sporting participation, cost of treatment and insurance cover)
- Do I need surgery now? Can I delay having surgery?

Important: This decision aid is not a substitute for advice from a health professional who should confirm your diagnosis. Radiculopathy, active infection, neoplastic disease, rheumatoid arthritis, thoracic myelopathy, trauma, ankylosing spondylitis and lumbar stenosis can all influence the decision-making process.

Disclosure: There was no funding to develop this tool. The developers of this decision aid include orthopaedic surgeons, physiotherapists, psychologists and occupational therapists. None of the developers will gain or lose anything based on the choices that people make.

Last reviewed: Update 10.5.2023. Developed by Andrew Gamble, Institute for Musculoskeletal Health, School of Public Health, The University of Sydney, NSW, Australia.

Which DCM category are you in?

- We can use the modified Japanese Orthopaedic Association (mJOA) score to classify DCM, and what treatment is most appropriate. [1]
- The scale ranges from 18 down to 0, with 18 being no symptoms (i.e., no spinal cord neurological symptoms) to 0 being severe symptoms (i.e., paralysed)*.

Scores are determined by the degree of difficulty when you:

- **0-5 Upper body movement**: button a shirt, use a spoon or move hands
- **0-7 Lower body movement:** walk, use stairs or move legs
- **0-3 Upper body:** feel or have increased pain with touch
- **0-3 Toileting:** have problems with toileting

Non-myelopathic (18)	Mild (15-17)	Moderate (12-14)	Severe (0-11)

*Scoring should be done with a health professional (lower number scores = worse DCM)

What are the recommended management options?

1. Non-surgical	2. DCM surgery

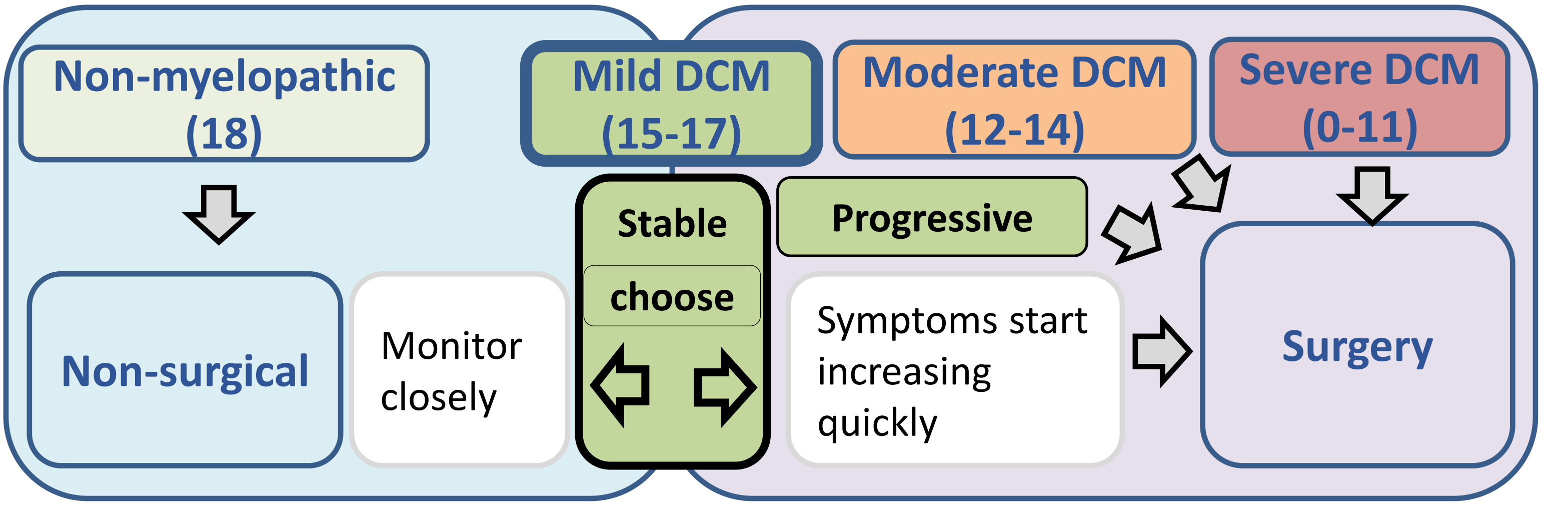

- **Non-myelopathic symptoms:** Monitor closely, learn about risks and know what signs and symptoms to look for in the future. [2] Ask if you have radiculopathy (a different nerve issue to DCM with no spinal cord compression).
- **Mild DCM: If stable,** non-surgical or surgery may be appropriate. **If progressive,** and nerve symptoms get worse or fail to improve - surgery is recommended.[2]
- **Moderate and Severe DCM:** Surgery is recommended. [2]

What do the management options involve?

Mild (15-17) | Progressive | Moderate (12-14) | Severe (0-11)

Surgery is recommended for people with:

- Mild DCM that is progressive
- Moderate DCM
- Severe DCM.[2]

Below are your options for stable mild DCM

1. Non-surgical management

Mild (15-17) | Stable

Trying non-surgical management before having surgery may be considered for people with **non-myelopathic or stable mild DCM. Milder DCM and a diagnosis before 6 months may mean you are more likely to feel benefits of non-surgical management.**[3]

Non-surgical management involves:

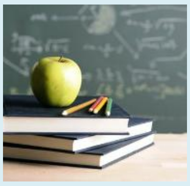

- Education
- Monitoring how quickly symptoms are changing
- Lifestyle and activity modification
- Structured exercise program to improve; strength, control and movement
- Medication or an injection

If you experience worsening of symptoms beyond 3-6 months despite doing your exercises and following advice, please talk to your health professional.

2. DCM surgery

Mild (15-17) | Stable

If your symptoms are quickly becoming worse with **mild DCM** and **non-surgical management** then please talk to your health professional about surgery. If your symptoms are getting worse (progressive), then surgery is recommended.

- Surgery involves a general anesthetic, making small cuts in the skin around your neck and using surgical tools to reduce spinal cord compression.
- There are different types of surgery e.g., approaching from the front or back of your neck.

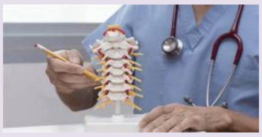

Following surgery, non-surgical management is needed over the next 3-6 months or longer depending on how you feel.

Comparing non-surgical management to DCM surgery

Mild DCM (15-17) stable	1. Non-surgical	2. DCM surgery
**How much will I improve?**	It is unknown how much you will improve. Mild DCM (stable) is more likely to improve with non-surgical management vs. mild DCM that is becoming progressive. [3]	
**Do I need surgery now?**	It is unknown if mild DCM will remain stable so the risk of surgery may be unnecessary. People who have worse DCM or had DCM more than 3 months may do better with surgery.[2]	
**How likely will I get worse over time?**	Depending on how bad your DCM is you may feel similar.[2]	
**What is the cost?**	May result in cost saving in the short-term	May result in cost saving in the long-term[2]
**What are the risks?**	After 1-year, you're 1.57x more likely to be admitted to hospital[4]	See risk of harms below
**Will I need delayed surgery? (mJOA 11-15)[2]**	23-54 in 100 people may still need DCM surgery	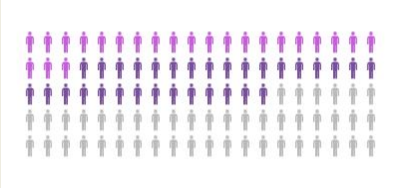

<u>**Surgery is recommended[2]**</u> (Average mJOA improvement)	**Mild DCM (15-17) Progressive**	**Moderate DCM (12-14)**	**Severe DCM (0-11)**
	7%	27%	27%

Combined risk of DCM surgery harms

**Minor and moderate harms**	Swallowing issues (dysphagia), loss of voice, increased pain and infection.<br>Some further spinal cord/nerve injury or worsening DCM[2]	14 in 100 people [2]<br>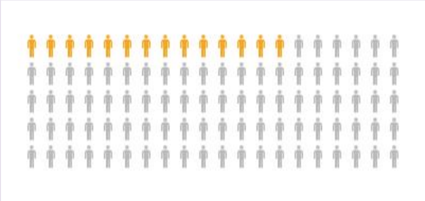
**Major harms**	Death, heart and lung issues, fracture or the need for another surgery[2]	

References:

1. Tetreault, L. et al,. (2017). Eur Spine J, 26(1), 78-84. https://doi.org/10.1007/s00586-016-4660-8
2. Fehlings, M. G. et al,. (2017). Global Spine J, 7(3 Suppl), 70s-83s. https://doi.org/10.1177/2192568217701914
3. Butler, M. B., et al. (2022). Global Spine Journal, 12(4), 638-645. https://doi.org/10.1177/2192568220961357
4. Rhee, J., et al,. (2017). Global Spine Journal 7, 35S–41S. doi:10.1177/2192568217703083
