# Supplementary material for: Development and acceptability of a patient decision aid for people with degenerative cervical myelopathy: an international mixed-methods study
Source: BMJ Open. 2026 Apr 3;16(4):e106337. doi: 10.1136/bmjopen-2025-106337 (PMC13052582; doi:10.1136/bmjopen-2025-106337)
Supplement: online supplemental file 10 [file bmjopen-16-4-s010.pdf]

# DEGENERATIVE CERVICAL MYELOPATHY

## Decision Aid: *Should I have surgery?*

All information in this decision aid should be discussed with a health professional familiar with Degenerative Cervical Myelopathy (e.g., doctor, neurologist, surgeon, physiotherapist)

## What is Degenerative Cervical Myelopathy?

**Degenerative = change over time** | **Cervical = neck related** | **Myelopathy = due to spinal cord damage**

- Degenerative Cervical Myelopathy is often called 'DCM'
- In DCM, nerve signals going between the brain and body via the spinal cord are disrupted due to bone and soft tissue changes that compress the spinal cord in the neck
- This ‘slowly, evolving spinal cord injury’ can impact muscles, feeling on the skin and organ function

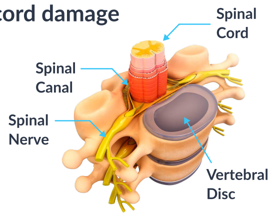


## How is Degenerative Cervical Myelopathy diagnosed?

### Changes And Symptoms Due To Spinal Cord Compression

- **What you feel** – pain, cramp, burning, numbness or pins and needles in the hands, arms or legs
- **Clumsiness or weakness** – dropping objects or less grip strength (e.g., trouble buttoning up a shirt)
- **Unsteady walking** – tripping or needing balance support

**Note:** Pain and stiffness is not always felt in the neck. Symptoms can be subtle and impact different parts of the body (e.g., bladder, bowel or sexual function changes).

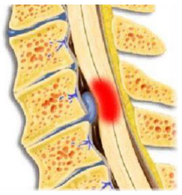

Pressure on the spinal cord

### Imaging

- MRI scans can show narrowing of the spinal canal (stenosis) or compression of the spinal cord

**Note:** While spinal cord compression can show on your scan, it may not be causing symptoms.

Symptoms and scan results both influence management decisions.

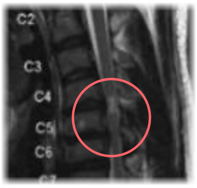


MRI scan

## What are the categories of Degenerative Cervical Myelopathy?

- The modified Japanese Orthopaedic Association (mJOA) scale is one way to classify the severity of DCM[1]
- Scores range from 18 (no spinal cord symptoms) to 0 (most severe)
- See page 2 for a link to calculate your score with a health professional who is familiar with DCM

| mJOA | Category | Description and management recommendations (in bold) |
|---|---|---|
| 18 | Non-myelopathic | This may be early DCM or a different nerve issue without spinal cord compression. **Learn about risks (page 3) and monitor with a health professional (page 4).[2]** |
| 15-17 | Mild DCM | No significant issues with walking or loss of muscle strength. No or mild changes in physical function. **Non-surgical management or DCM surgery may be appropriate.[2]** |
| 12-14 | Moderate DCM | Your walking may feel unstable. You may have difficulty with hand coordination. **Timely surgery is recommended.[2]** |
| 0-11 | Severe DCM | Walking may be difficult. You may need a walking aid. You are likely to have significant muscle weakness or numbness. **Urgent surgery is recommended.[2]** |

## Which Degenerative Cervical Myelopathy category are you in when using the mJOA tool?

- The mJOA score rates difficulty using your arms, legs and toileting[1]
- Access the mJOA tool via the QR code or [Assessment Scales - MYELOPATHY.ORG]

Date: ____________ Current mJOA total score: ____________

- ○ Non-myelopathic (18)
- ○ Mild DCM (15-17)
- ○ Moderate DCM (12-14)
- ○ Severe DCM (0-11)

## What is recommended?

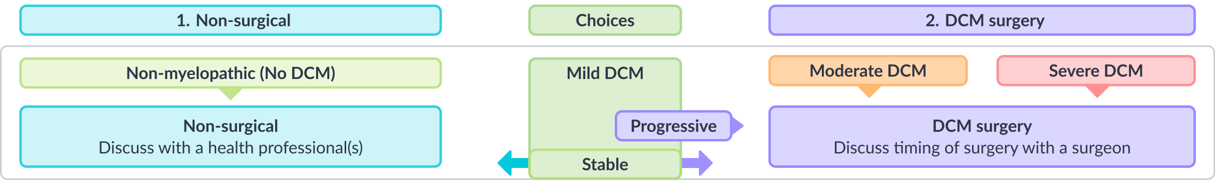


- People with mild (stable) DCM have the option to choose between non-surgical management or DCM surgery
- Treatment for people with mild DCM is decided with a health professional and based on symptoms, imaging and personal preferences
- Researchers are still learning about how well nerves can heal on their own

### 1 NON-SURGICAL MANAGEMENT

This is an option for people with DCM whose symptoms remain stable. The strategies below can be used without surgery. They can also be used before or after surgery with the aim to improve surgery outcomes.

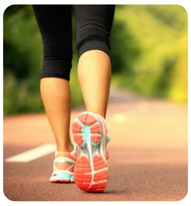

**A health professional can guide you with:**

- Lifestyle and activity modification (e.g., physical activity levels or avoiding aggravating positions like looking up for a long time to cut hedges)
- Planning an exercise program to improve strength, movement and balance
- Self-management (e.g., pain, mindfulness and good sleep)
- Learning about DCM and monitoring symptoms (e.g., test grip strength and balance, discuss if you need a follow up MRI or use a diary to note key changes)
- Caring for your mental health and wellbeing. Join the the DCM social group via the QR code or

[Myelopathy.org Support Group | Facebook]

### 2 DCM SURGERY

This is an option for people with DCM whose symptoms do not remain stable or are significant.[3] Surgery aims to slow the progression of DCM and can improve some symptoms for some people.

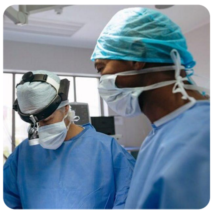

- A surgical procedure is used to reduce spinal cord compression under general anaesthetic
- Surgery is performed either from the front or back of the neck. It involves joining bones of the spine together or inserting screws and plates to stabilise the spine
- Your surgeon will advise you on the type of surgery that is best for you (e.g., ACDF-Anterior Cervical Discectomy and Fusion, posterior cervical decompression)

**Following surgery:**

- Rehabilitation with other health professionals can help recovery (e.g., allied health professionals such as physiotherapists)
- Healing and adjusting to symptoms can occur over the next 1-2 years and beyond[3]

## What are potential harms of non-surgical management vs DCM surgery?

- This page shows estimates from the best available evidence from 5 studies including 1048 people with DCM.
- The results below are based on averages. We cannot tell if you will benefit from a particular treatment or experience a harm.

### 1 NON-SURGICAL MANAGEMENT

### 2 DCM SURGERY

10 more people with **mild-moderate DCM** out of 100 get worse by at least 1 point on the mJOA scale with non-surgical management at 10 years[4]:

**Key:** People who get worse

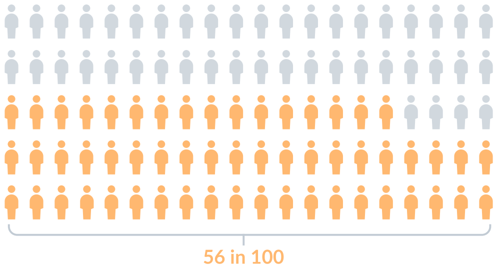


vs

**Key:** People who get worse

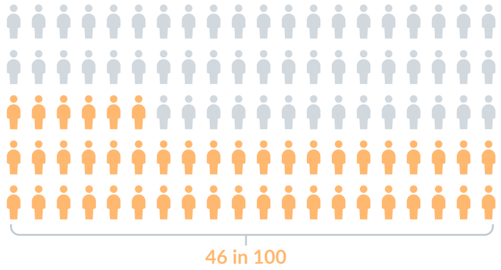


People with **mild DCM** managed non-surgically often get worse over time[5]:

**Key:** People who get worse

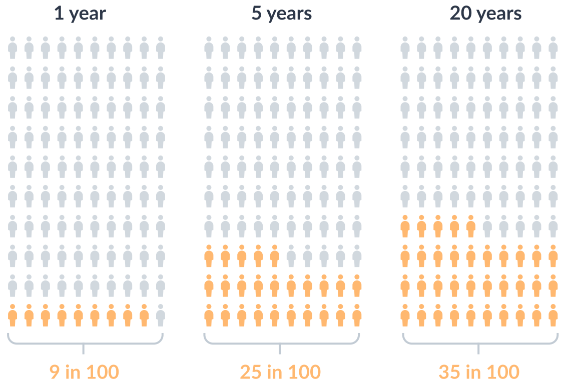


After surgery, 14 people out of 100 may experience harms, regardless of DCM severity[2]:

**Key:** People who experience harm

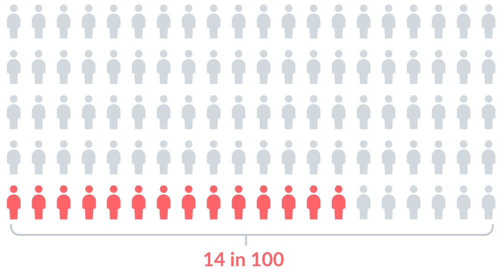


**Examples of mild-moderate harms:** Pain 6%, swallowing issues 2%, infection 2%, worse DCM 1%
**Examples of serious harms:** Death or stroke 0.3%, more surgery 1%, fracture 2%, heart and lung issues 3%

## What are potential benefits of non-surgical management and DCM surgery?

### 1 NON-SURGICAL MANAGEMENT

29 people with **mild DCM** out of 100 report benefits of physiotherapy[6]:

**Key:** People who benefit from physiotherapy

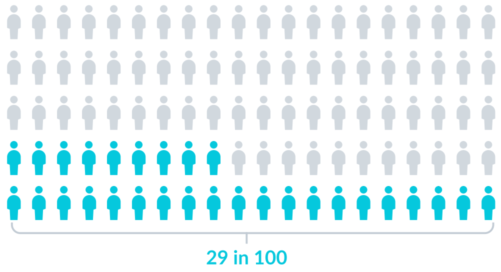


### 2 DCM SURGERY

Between 23 and 38 people with **mild-moderate DCM** out of 100 who start non-surgical management may need surgery at 2-7 years[7]:

**Key:** People who need surgery

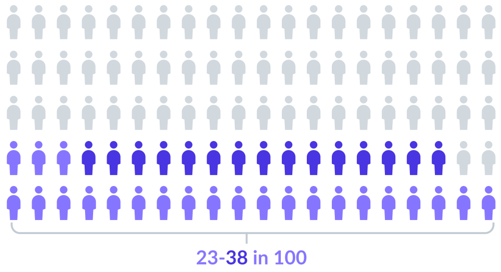

## Questions for when you talk with a health professional...

- What are the benefits and harms of surgery and non-surgical management for me?
- What do you offer as non-surgical management and how often will I be reassessed?
- Do I need surgery now? If I delay surgery, when should I return for follow up?
- What are my expected outcomes post-treatment? What will rehabilitation look like?
- What else do I need to consider? (general health e.g., age, weight; work; leisure activities e.g., gym, cycling)

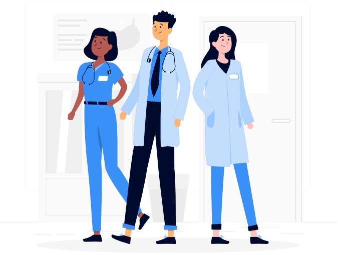

**Ask yourself..**

1. Is your quality of life impaired by DCM?
2. Do you have significant pain?

People with low quality of life or neck pain may be candidates for DCM surgery.[8]

**Monitor key changes with a health professional and mention if...**

- Your walking changes or you become unsteady (e.g., using a handrail)
- Your hands become clumsy (e.g., buttoning a shirt or writing)
- You develop worsening neck or arm pain

Follow up date to review with a healthcare professional: ______________________

## Where can I find more information?

Myelopathy.org https://myelopathy.org/ (includes a UK helpline)

Spinal Injuries Association UK https://spinal.co.uk/

Spinal Injuries Association Australia https://scia.org.au/

**Important information:** This decision aid is not a substitute for advice from a health professional who should confirm your diagnosis. Having other conditions such as radiculopathy, an infection, neoplastic disease, rheumatoid arthritis, thoracic myelopathy, trauma, ankylosing spondylitis and lumbar stenosis can all influence the decision-making process.

**Disclosure:** The National Health and Medical Research Council (NHMRC) provided funding to develop this tool but had no involvement in the development process. The developers of this decision aid include physiotherapists, surgeons, neurologists, doctors, specialists and a chiropractor. None of the developers will gain or lose anything based on the choices that people make.

**Last reviewed:** 21.1.26. Updated due 21.1.28.

**Lead developer:** Andrew R Gamble, Institute for Musculoskeletal Health, The University of Sydney, NSW, Australia.

**References:**

1. Tetreault, L. et al,. (2017) Eur Spine J. 26(1), 78-84.
2. Fehlings, M. G. et al,. (2017) Global Spine J. 7(3), 70s-83s.
3. Evaniew et al,. (2023) Spine J. 48(5):310-320.
4. Kadaňka, Z. et al (2011) Eur Spine J. 20(9):1533-8.
5. Sarraj M., et al,. (2024) Spine J. 24(1):46-56.
6. Butler, M. B., et al,. (2022) Global Spine J. 12(4), 638-645.
7. Tetreault, L. et al,. (2017) Global Spine J. 7(3):42s-52s.
8. Khosravi S., et al,. (2023) Global Spine J. 14(2):697-706.

The University of Sydney · Institute for Musculoskeletal Health · NMSK Research · SHAREDDCM · Sydney Health Literacy Lab · WISER HEALTHCARE
