# Supplementary material for: Development and acceptability of a patient decision aid for people with degenerative cervical myelopathy: an international mixed-methods study
Source: BMJ Open. 2026 Apr 3;16(4):e106337. doi: 10.1136/bmjopen-2025-106337 (PMC13052582; doi:10.1136/bmjopen-2025-106337)
Supplement: online supplemental file 11 [file bmjopen-16-4-s011.docx]

| Supplementary File 11: International Patient Decision Aid Standards checklist  (IPDASi v4.0) | |
| --- | --- |
| **Qualifying criteria** | **Answer** |
| 1. The patient decision aid describes the health condition or problem (treatment, procedure, or investigation) for which the index decision is required. | Yes |
| 2. The patient decision aid explicitly states the decision that needs to be considered (index decision). | Yes |
| 3. The patient decision aid describes the options available for the index decision. | Yes |
| 4. The patient decision aid describes the positive features (benefits or advantages) of each option. | Yes |
| 5. The patient decision aid describes the negative features (harms, side effects, or disadvantages) of each option. | Yes |
| 6. The patient decision aid describes what it is like to experience the consequences of the options (e.g., physical, psychological, social). | Yes |
| **Certification criteria** | **Answer** |
| 1. The patient decision aid shows the negative and positive features of options with equal detail (e.g., using similar fonts, sequence, presentation of statistical information). | Yes |
| 2. The patient decision aid (or associated documentation) provides citations to the evidence selected. | Yes |
| 3. The patient decision aid (or associated documentation) provides a production or publication date. | Yes |
| 4. The patient decision aid (or associated documentation) provides information about the update policy. | Yes |
| 5. The patient decision aid provides information about the levels of uncertainty around event or outcome probabilities (e.g., by giving a range or by using phases such as ‘‘our best estimate is . . .’’). | Yes |
| 6. The patient decision aid (or associated documentation) provides information about the funding source used for development. | Yes |
| 7. The patient decision aid describes what the test is designed to measure. | N/A |
| 8. If the test detects the condition or problem, the patient decision aid describes the next steps typically taken. | N/A |
| 9. The patient decision aid describes the next steps if the condition or problem is not detected. | N/A |
| 10. The patient decision aid has information about the consequences of detecting the condition or disease that would never have caused problems if screening had not been done (lead time bias). | N/A |
| **Quality criteria** | **Answer** |
| 1. The patient decision aid describes the natural course of the health condition or problem, if no action is taken (when appropriate). | Yes |
| 2. The patient decision aid makes it possible to compare the positive and negative features of the available options. | Yes |
| 3. The patient decision aid provides information about outcome probabilities associated with the options (i.e., the likely consequences of decisions). | Yes |
| 4. The patient decision aid specifies the defined group (reference class) of patients for whom the outcome probabilities apply. | Yes |

| 5. The patient decision aid specifies the event rates for the outcome probabilities | Yes |
| --- | --- |
| 6. The patient decision aid allows the user to compare outcome probabilities across options using the same time period (when feasible). | Yes |
| 7. The patient decision aid allows the user to compare outcome probabilities across options using the same denominator (when feasible). | Yes |
| 8. The patient decision aid provides more than 1 way of viewing the probabilities (e.g., words, numbers, and diagrams). | Yes |
| 9. The patient decision aid asks patients to think about which positive and negative features of the options matter most to them (implicitly or explicitly). | Yes |
| 10. The patient decision aid provides a step-by step way to make a decision. | Yes |
| 11. The patient decision aid includes tools like worksheets or lists of questions to use when discussing options with a practitioner. | Yes |
| 12. The development process included a needs assessment with clients or patients. | Yes |
| 13. The development process included a needs assessment with health professionals. | Yes |
| 14. The development process included review by clients/patients not involved in producing the decision support intervention. | Yes |
| 15. The development process included review by professionals not involved in producing the decision support intervention. | Yes |
| 16. The patient decision aid was field tested with patients who were facing the decision. | Yes |
| 17. The patient decision aid was field tested with practitioners who counsel patients who face the decision. | Yes |
| 18. The patient decision aid (or associated documentation) describes how research evidence was selected or synthesized. | Yes |
| 19. The patient decision aid (or associated documentation) describes the quality of the research evidence used. | Yes |
| 20. The patient decision aid includes authors’/developers’ credentials or qualifications. | Yes |
| 21. The patient decision aid (or associated documentation) reports readability levels (using 1 or more of the available scales). | Yes |
| 22. There is evidence that the patient decision aid improves the match between the preferences of the informed patient and the option that is chosen. | No* |
| 23. There is evidence that the patient decision aid helps patients improve their knowledge about options’ features. | No* |
| 24. The patient decision aid includes information about the chances of having a true-positive test result. | N/A |
| 25. The patient decision aid includes information about the chances of having a true-negative test result. | N/A |
| 26. The patient decision aid includes information about the chances of having a false-positive test result. | N/A |
| 27. The patient decision aid includes information about the chances of having a false-negative test result. | N/A |
| 28. The patient decision aid describes the chances the disease is detected with and without the use of the test. | N/A |

N/A: not applicable.

*We plan to evaluate the decision aid in a randomised controlled trial.
