# Supplementary material for: Development and acceptability of a patient decision aid for people with degenerative cervical myelopathy: an international mixed-methods study
Source: BMJ Open. 2026 Apr 3;16(4):e106337. doi: 10.1136/bmjopen-2025-106337 (PMC13052582; doi:10.1136/bmjopen-2025-106337)
Supplement: online supplemental file 13 [file bmjopen-16-4-s013.docx]

Supplementary file 13: Reasons for not implementing feedback for each section of the decision aid

| Themes | Sub themes | Feedback | Reason for not implementing the feedback |
| --- | --- | --- | --- |
| 2. Modify the decision aid to enhance understanding for people with DCM | 2.1  Constructive feedback on the content | Patient-participants |  |
|  |  | Some feedback indicated that certain content may be too complex. | Some content suggested to be too complex was necessary for accuracy of the decision aid. The decision aid also includes prompts to discuss content with a health professional. |
|  | 2.2 Simplify the language used | Patient-participants |  |
|  |  | Some patient-participants suggested some language was too technical. | Some technical terms are necessary for accuracy of the decision aid. |
|  | 2.3 Modify the information included | Patient-participants |  |
|  |  | Suggestions were made by patient-participants to remove less relevant information. For example some pictures could be changed to be more relevant to the context.  Suggestions were made to restructure the information so the decision portion of the decision aid was on the first page.  A suggestion was made to provide the full Modified Japanese Orthopaedic Association scale rather than just the QR code for people with DCM who are less technology literate. | Information has been prioritised, but some details remained essential for a complete understanding.  Information was restructured over the development process, but researchers felt it was important to have a description of DCM before the decision portion of the decision aid.  Researchers felt that the QR code simplified the decision aid and the decision aid is designed to be used with a health professional who could guide use of the QR code if required. |
|  |  | Health Professional-participants |  |
|  |  | Health professional-participants suggested to add differential diagnosis information and additional information about scans. | The decision aid includes information about diagnosis but additional information about differential diagnosis should be discussed with a health professionals to avoid confusion. |
|  | 2.4 Modify pictures and graphics | Patient-participants |  |
|  |  | Suggestions were made by people with DCM to adjust visuals for better clarity. | Not all suggested changes were accommodated due to design constraints. |
|  |  | Health Professional-participants |  |
|  |  | Some health professional-participants suggested making pictures more relevant to the context. | The pictures included were chosen to enhance understanding for people with DCM. |
| 3. Improving awareness of DCM and acceptability of the decision aid | 3.1 Clarify the purpose of the decision aid | Patient-participants |  |
|  |  | There was a need for further explanation of the decision aid’s purpose. | We decided to focus on using headings to describe the purpose of the decision aid to avoid confusion. For example, ‘Should I have surgery?’. |
|  | 3.2 Increasing awareness of DCM and evidence-based management | Patient-participants |  |
|  |  | Requests were made for more background on DCM and management strategies. | Feedback consistently suggested the decision aid should be simplified so it was decided that additional detail was unnecessary. |
|  |  | Health Professional-participants |  |
|  |  | Health professional-participants sought more comprehensive details on DCM and evidence-based management to support their guidance of care of people with DCM. | The need to remain concise limited the depth of additional information that could be included. |
|  | 3.3 Increasing the potential for practical implementation of the decision aid | Patient-participants |  |
|  |  | Suggestions were made to make the aid more applicable to real-life scenarios. | We avoided including case studies as frequent feedback suggested to keep the decision aid concise. |
|  |  | Health Professional-participants |  |
|  |  | Health professional-participants requested changing the format of the Modified Japanese Orthopaedic Association scale to improve practical application. | Certain recommendations, such as modifying the Modified Japanese Orthopaedic Association scale were beyond the current scope of the aid. |
|  | 3.4 Determining how to best include the Modified Japanese Orthopaedic Association (mJOA) scale and encourage use of outcome measures | Patient-participants |  |
|  |  | Requests were made to integrate the Modified Japanese Orthopaedic Association scale more effectively or change how scoring was presented | We were unable to change how the Modified Japanese Orthopaedic Association scale scoring was presented. |
|  |  | Health Professional-participants |  |
|  |  | Health professional-participants suggested that the Modified Japanese Orthopaedic Association scale should be more prominently featured to guide clinical decisions. | Due to feedback about making the decision aid more concise we decided not to include the whole Modified Japanese Orthopaedic Association scale. |
| 4. Highlight variations in symptoms and promote individual management | 4.1 Acknowledge the variation of DCM symptoms and individual circumstances | Health Professional-participants |  |
|  |  | Health professional-participants emphasised the importance of recognising individual variations in symptoms. | To keep the decision aid concise we avoided including every example of symptoms mentioned in interviews. |
|  | 4.2 Promote the use of multiple objective tools to guide timely management | Patient-participants |  |
|  |  | Suggestions were made to include various tools for decision-making. | Not all decision-making tools could be included due to space and focus constraints. |
|  |  | Health Professional-participants |  |
|  |  | Health professional-participants recommended including various objective tools to further guide decision making. | To keep the decision aid concise, not all suggestions could be incorporated. |
| 5. Create realistic treatment expectations | 5.1 Awareness of non-surgical and surgical management aims for people with DCM | Patient-participants |  |
|  |  | There was a need to clarify goals for non-surgical and surgical treatments. | Some feedback on goals was too case-specific for broad application to be included. |
|  |  | Health Professional-participants |  |
|  |  | Health professional-participants requested clearer goals for both non-surgical and surgical management. | Goals should be tailored to each person with DCM, so were not specific in the decision aid. |
|  | 5.2 Clarify guidelines for rehabilitation and potential for spinal cord healing | Patient-participants |  |
|  |  | There was a need for clear rehabilitation and healing guidelines. | Due to the limited availability of evidence to guide rehabilitation for DCM and spinal cord healing we were unable to provide further detail. |
|  |  | Health Professional-participants |  |
|  |  | Health professional-participants requested detailed guidelines on rehabilitation and spinal cord healing. | Due to the limited availability of evidence to guide rehabilitation for DCM and spinal cord healing we were unable to provide further detail. |
|  | 5.3 Highlight challenges to access and affordability of care for people with DCM | Patient-participants |  |
|  |  | Concerns were raised about access to and affordability of care. | The decision aid is designed to be used globally. It can be adapted to incorporate specifc access and affordability to care ifnromation for different countries in the future. |
|  |  | Health Professional-participants |  |
|  |  | Health professional-participants stressed the need to address barriers to access and affordability. | Providing practical solutions around access and affordability of care was outside the scope of the decision aid due to differences between countries and to keep the decision aid more concise. |

DCM, Degenerative Cervical Myelopathy, QR, quick response
