# Supplementary material for: Development and acceptability of a patient decision aid for people with degenerative cervical myelopathy: an international mixed-methods study
Source: BMJ Open. 2026 Apr 3;16(4):e106337. doi: 10.1136/bmjopen-2025-106337 (PMC13052582; doi:10.1136/bmjopen-2025-106337)
Supplement: online supplemental file 14 [file bmjopen-16-4-s014.docx]

Supplementary file 14: Interview themes and subthemes, and example quotes

| Themes | Sub themes | Quotes |
| --- | --- | --- |
| 1. Positive feedback on the decision aid | 1.1. Positive feedback on the content | Patient-participants |
|  |  | *"I like the graphics that you've got on there. And I like the fact that it's putting the decision-making back with the patient but giving them information." (F, 51-60, patient)*  *"I wonder if I had had access to this clarity of information, if it would have helped me to think more logically at the time" (F, 61-70, patient)*  *"Clearly listed in everyday language" (F, 41-50, patient)* |
|  |  | Health professional-participants |
|  |  | *"I mean, I think this is a really nice piece of piece of work developing something that, you know, is a, essentially, I would call it a shared decision-making tool." (F, 41-50, physiotherapist)*  *"If neurologists had access to data like this then we would be better suited to help them or help triage them appropriately" (F, 31-40, neurologist)* |
|  | 1.2 Positive feedback on the design | Patient-participants |
|  |  | *""I really liked this, everything was presented objectively." (F, 61-70, patient)*  *"Everything was clear, I've been through the whole document, and I find it very clear, very easy to follow." (F, 61-70, patient)* |
|  |  | Health professional-participants |
|  |  | *"The layout is clean and easy to follow. It’s not overwhelming like other medical guides I’ve seen." (F, 41-50, physiotherapist)* |
| 2. Modify the decision aid to increase understanding for people with DCM | 2.1. Constructive feedback on the content | Patient-participants |
|  |  | *"Some of the medical jargon was difficult to understand without prior knowledge." (F, 61-70, patient)*  *"Again, that first bit, it steers towards surgery... I feel that this still seems to stitch things in that the surgery is recommended for people with and I don't think it depends on the timeline." (F, 61-70, patient)* |
|  |  | Health professional-participants |
|  |  | *"The document itself is dense with a lot of material but there are ways to simplify it" and "you could use more lay definitions" (M, 61-70, neurosurgeon)*  *"Needs to be more patient friendly" (M, 21-30, physiotherapist)* |
|  | 2.2 Constructive feedback on the design | Patient-participants |
|  |  | *"Very text heavy, needs more white space" (F, 51-60, patient)*  *“...surgical cuts...I would have just said surgery from the front or the back of the neck...surgical procedure to reduce spinal cord compression.” (F, 71-80, patient)* |
|  |  | Health professional-participants |
|  |  | *"I think we need you to modify this" (F, 21-30-, occupational physician)*  *"It’s quite text-heavy. More white space and bullet points would make it less daunting." (F, 41-50, physiotherapist)* |
|  | 2.3 Simplify the language used | Patient-participants |
|  |  | *"Simplifying the language and defining each part of DCM would make the information more accessible." (F, 61-70, patient)*  *"Breaking down complex terms into layman's language could help patients better understand their condition." (F, 61-70, patient)*  *"For lay people, you may want to simplify it a little bit." (M, 71-80, patient)*  *"I’m concerned in our increasingly sound bite-driven society of, you know, 150 characters or less, some of the info is going to get lost in the weeds." (M, 31-40, patient)* |
|  |  | Health professional-participants |
|  |  | *"Use lay language for the reader" (M, 41-50, neurosurgeon)*  *"You could use more lay definitions" (M, 61-70, neurosurgeon)* |
|  | 2.4 Modify how statistics are presented | Patient-participants |
|  |  | *"Oh, perhaps you could add pictures to it like the other one." (F, 51-60, patient)*  *"One of the challenging things in deciphering this information is that it's dealing with different time scales... you have information for non-surgical management at one year, five years, and surgery at three months, one year." (M, 31-40, patient)* |
|  |  | Health professional-participants |
|  |  | *"I did wonder like whether a QR code would be helpful, that could take them through to either a website or an option for larger texts or different languages. Which would be the other thing, I don't know if there's a if you thought about other language versions, but certainly putting a QR code that then gives them because I think when I read this through" (F, 41-50, physiotherapist)*  *"The statistics should be presented in a more visual format. Graphs or charts could make them easier to understand." (F, 41-50, physiotherapist)* |
|  | 2.5 Modify the information included | Patient-participants |
|  |  | *"And it's not just healing, it's adapting to the fused vertebra as well, depending on how much you know, range of movement, you have post-surgery." (F, 41-50, patient)*  *"The symptoms of DCM can vary widely. Early symptoms often include loss of fine motor skills and balance issues, as you mentioned. It’s useful to document specific experiences, such as difficulty with buttoning shirts or navigating stairs." (F, 41-50, patient)*  *"It would be interesting to know if there's been a study that has looked at perceived benefit of surgery in people with mild DCM... So then you can have, like, what is the perceived benefit of both." (M, 31-40, patient)* |
|  |  | Health professional-participants |
|  |  | *"Including more information on the diverse symptoms of DCM would make it more relatable to different patients." (F, 41-50, physiotherapist)* |
|  | 2.6 Modify the pictures and graphics | Patient-participants |
|  |  | *"Or you might have room for you know, if you change the picture of the lady walking to a different shape, you might have room for some graphics to put in, you know, a tablet and a pillow or an or mattress." (F, 41-50, patient)*  *"The picture on the non-surgical one looks a bit too laid back and relaxed. I'd like to see that somebody can continue to be active there, rather than just sit by the bill and read a book." (F, 61-70, patient)* |
|  |  | Health professional-participants |
|  |  | *"The graphics need to be more illustrative and relevant to the text. They should help in explaining the content better." (F, 41-50, physiotherapist)*  *"Possibly use colour codes (green, yellow, red) to indicate severity levels." (F, 21-30, occupational physician)*  *"Consider breaking down complex information into simpler chunks." (F, 21-30, occupational physician)* |
| 3. Improving awareness of DCM and acceptability of the decision aid | 3.1 Clarify the purpose of the decision aid | Patient-participants |
|  |  | *"I like that all information should be discussed with a health professional" (F, 41-50, patient)*  *"I think what potentially we should have is a better explanation of the MJOA. And also explain how it's determined." (F, 51-60, patient)* |
|  |  | Health professional-participants |
|  |  | *"I like that it should be discussed with a health professional - people google or talk…" (M, 51-60, neurosurgeon)*  *"It should be clearer that this decision aid is meant to be used alongside a health professional to avoid causing unnecessary panic." (F, 41-50, physiotherapist)* |
|  | 3.2 Awareness of DCM and evidence-based management | Patient-participants |
|  |  | *"I didn’t know what it was until I found out I had it and I even have trouble explaining it to friends now so I just say I have a spinal cord injury" (F, 41-50, patient)*  *"5-7 years then deterioration and over really poorly diagnosed area" “overseas trained doctors sometimes have little knowledge of it” (F, 71-80, patient)* |
|  |  | Health professional-participants |
|  |  | *"Awareness is an issue in primary care" "thresholds can be subjective and people need to get the right information, can’t all talk to surgeons" (M, 21-30, physiotherapist)*  *"People don’t seek help in parts of Scotland until very late as they think it is normal aging and can be only 51 years old" (M, 41-50, neurosurgeon)* |
|  | 3.3 Increase the potential for practical implementation of the decision aid | Patient-participants |
|  |  | *“...learning about DCM and monitor your symptoms with your health professional.” (F, 71-80, patient)* |
|  |  | Health professional-participants |
|  |  | *"it's probably for me going back to where you think this is going to be the most useful. Because I think if you are trying to develop something for everybody, you might struggle." (F, 41-50, physiotherapist)*  *"So in primary care, we would say, in the UK, where this can be delivered by a healthcare professional, you can start to have those conversations, as you're referring a patient on almost to get them ready for that surgical conversation, then that will be brilliant. From my perspective, when patients come to us, they are just not expecting the conversation about surgery. And the MJOA hasn't even been done, they haven't been categorised that when they get to us, and we say, okay, surgery is your option, they almost panic” (F, 41-50, physiotherapist)* |
|  | 3.4 Determine how to best include the mJOA scale and encourage use of outcome measures | Patient-participants |
|  |  | N/A |
|  |  | Health professional-participants |
|  |  | *“We don’t need to teach patients how to calculate the mJOA… (M, 61-70, neurosurgeon)*  *"Including quality of life scales instead of the full mJOA might be more useful for guiding management decisions." (F, 41-50, physiotherapist)* |
| 4. Highlight variations in symptoms and promote individual management | 4.1 Acknowledge the variation of DCM symptoms and individual circumstances | Patient-participants |
|  |  | *"You might just want to put in balance issues there as well. So you've got clumsiness difficult. Yeah. She's walking in, but you haven't got a lot of other... I've got quite a lot of balance issues." (F, 51-60, patient)*  *"The clumsiness and tripping over were the main symptoms for me." (F, 51-60, patient)* |
|  |  | Health professional-participants |
|  |  | *"Pain in the arms can occur but the disease is more focused on lack of coordination and unsteady gait" (M, 41-50, neurosurgeon)*  *"Early symptoms look like this, you know, get an MRI scan done, they're covered by Medicare." (F, 21-30, occupational physician)* |
|  | 4.2 Promote the use of multiple objective tools to guide timely management | Patient-participants |
|  |  | *"Let them know if your experience worsening, worsening of symptoms despite following medical advice." (F, 41-50, patient)* |
|  |  | Health professional-participants |
|  |  | *"I talk to them about symptoms worsening or the impact of their life, but the score is just where they stand" (M, 41-50, neurosurgeon)*  *"Tools like the mJOA should be used to highlight the urgency and guide the management of the condition." (F, 41-50, physiotherapist)* |
|  | 4.3 Encourage the appropriate use of shared decision making | Patient-participants |
|  |  | *"Your surgeon will help you decide on the type of surgery" (F, 41-50, patient)*  *“...talk to a health professional about potential surgery...your symptoms may not improve, or, you know, may only be, you know, minimal, but it's the aim is to prevent, or to try and stop symptom progression.” (F, 71-80, patient)*  *"Asking the patient, 'are you staying the same, getting better, getting worse'... might cause them to take a moment to reflect on what their state is before thinking about what these categories are." (M, 31-40, patient)* |
|  |  | Health professional-participants |
|  |  | *"Treatment is determined by symptoms and image findings" (M, 51-60, orthopaedic surgeon)*  *"This should be an individualised discussion specific to that patient (M, 21-30, physiotherapist)*  *"It can be a silent bomb sometimes you know" (M, 41-50, neurosurgeon)*  *Not only the mJOA that will dictate the treatment is good you have mentioned (F, 31-40, neurologist)* |
| 5. Create realistic treatment expectations | 5.1 Awareness of non-surgical and surgical management aims for people with DCM | Patient-participants |
|  |  | *"No pain no gain common – not always the case particularly the case with the neck" (F, 41-50, patient)*  *"It’s important to clearly state that non-surgical management is a viable option and can be effective for many individuals." (F, 61-70, patient)* |
|  |  | Health professional-participants |
|  |  | *"It is a degenerative condition, so management isn't a cure" (F, 41-50, patient)*  *"The importance of setting a realistic expectation and the fact that people aren't necessarily cured by this." (M, 51-60, general practitioner)*  *Careful with language - is it a slowly progressive spinal cord injury vs scary slow motion spinal cord injury (M, 21-30, physiotherapist)*  *"Quite often the expectations sign off for surgery is that it fixes everything and not realistic and not what they hear even if it has been said" (M, 41-50, rehabilitation and pain specialist)* |
|  | 5.2 Clarify guidelines for rehabilitation and potential for spinal cord healing | Patient-participants |
|  |  | *“Post op care and rehab makes a huge difference – persistence with exercises and recommendations from OT” (F, 41-50, patient)*  *"I started doing step training, just to try and get my balance. And then I was able to do it with weights, you know how you can stay active?" (F, 61-70, patient)* |
|  |  | Health professional-participants |
|  |  | *"Rehabilitation is important post-surgery, but type and timing should be personalised. People may not have insurance or recover well with home exercise" (M, 61-70, neurosurgeon)*  *"Detailed recommendations for rehabilitation, including physiotherapy, should be clarified." (F, 41-50, physiotherapist)* |
|  | 5.3 Highlight challenges to access and affordability of care for people with DCM | Patient-participants |
|  |  | *“So in the NHS, no rehabilitation is provided nothing. After my first operation, I was so traumatised, I was so traumatised after surgery. And adapting can take time. (F, 71-80, patient)*  *“...access and affordability of treatment options can vary significantly between public and private healthcare systems.” (F, 71-80, patient)*  *“...many patients struggle with the cost of follow-up appointments and repeat scans, which are often necessary for ongoing management.” (F, 71-80, patient)*  *"Surgery booking takes time, so the priority should go to people with fast-progressing symptoms." (M, 71-80, patient)* |
|  |  | Health professional-participants |
|  |  | *"Information on the affordability and accessibility of treatments, including the role of primary practitioners, is needed." (F, 41-50, physiotherapist)* |
| 6. Facilitate equitable access to care and active management strategies | 6.1 Encourage monitoring of DCM symptoms with a health professional | Patient-participants |
|  |  | *"It's important to monitor symptoms, especially on your worst days, as they can vary day to day." (F, 71-80, patient)*  *“...monitoring is about...self-awareness on the patients.” (F, 71-80, patient)* |
|  |  | Health professional-participants |
|  |  | *"Non-myelopathic may not need to be monitored closely but rather come back if they develop changes" (M, 41-50, neurosurgeon)*  *"They may not need to see anyone, but they need to know the risk factors and when to come back” (M, 41-50, neurosurgeon)*  *"Patients can have this progressive condition that is silent, that they don't have any pain or there's just steadiness and they think it's old age." (F, 21-30, occupational physician)* |
|  | 6.2 Participation in management and creating foundations for on-going support | Patient-participants |
|  |  | *"Engage in regular, tailored physical therapy sessions to maintain muscle strength, improve balance, and enhance coordination. Focus on exercises that can be done safely under professional supervision." (F, 61-70, patient)*  *"Promoting active participation in management and building a support system for patients is crucial." (F, 41-50, physiotherapist)* |
|  |  | Health professional-participants |
|  |  | *"The key with these patients is the psychosocial factors which need to be delt with" (M, 31-40, orthopaedic surgeon)*  *"I talk to them about symptoms worsening or the impact of their life, but the score is just where they stand" (M, 41-50, neurosurgeon)* |
|  | 6.3 Acknowledge the adjustments to living with DCM and benefits of support groups | Patient-participants |
|  |  | *"I was mild when they looked at me because I could walk into the room and if I could articulate everything, and I was still functioning at a level, where I was caring for my children, I was still driving. But I knew compared to what my function was in 2020, that I was vastly different." (F, 41-50, patient)* |
|  |  | Health professional-participants |
|  |  | *"Providing information on support groups and realistic timeframes for adjusting to life with DCM would be very helpful." (F, 41-50, physiotherapist)* |
|  | 6.4 Promote a positive mindset for the future management of people with DCM | Patient-participants |
|  |  | *"My neurosurgeon saved my life... he has pointed me in the direction of how to best approach non-surgical routes." (F, 61-70, patient)*  *“...highlighting success stories of individuals who have managed their DCM well can provide hope and motivation.” (F, 71-80, patient)* |
|  |  | Health professional-participants |
|  |  | *"Incorporating positive messages throughout the decision aid could help boost patients' morale and outlook on managing DCM." (F, 41-50, physiotherapist)* |

F, Female, M, Male, DCM, Degenerative cervical myelopathy, QR, quick response, UK, United Kingdom, mJOA, modified Japanese Orthopaedic Association, NHS, National Health Scheme, OT, Occupational Therapist
