# Supplementary material for: Development and acceptability of a patient decision aid for people with degenerative cervical myelopathy: an international mixed-methods study
Source: BMJ Open. 2026 Apr 3;16(4):e106337. doi: 10.1136/bmjopen-2025-106337 (PMC13052582; doi:10.1136/bmjopen-2025-106337)
Supplement: Supplementary file 15 [file bmjopen-2025-106337.reviewer_comments.pdf]

PEER REVIEW HISTORY

BMJ Open publishes all reviews undertaken for accepted manuscripts. Reviewers are asked to complete a checklist review form and are provided with free text boxes to elaborate on their assessment. These free text comments are reproduced below.

ARTICLE DETAILS

Title (Provisional)

Development and acceptability of a patient decision aid for people with degenerative cervical myelopathy: an international mixed-methods study

Authors

Gamble, Andrew R; Anderson, David B; McKay, Marnee Jean; Davies, Benjamin; Macpherson, Sophie; Van Gelder, James; Hoffmann, Tammy; McCaffery, Kirsten; Stevens, Samuel; Ammendolia , Carlo; Chauhan, Rohil V; Zipser, Carl; Boerger, Timothy F; Tetreault, Lindsay; Fehlings, Michael G.; Dustan, Elanor; Nugent, Carolyn; Holmgren, Helen; Demetriades, Andreas; Lantz, Justin; Dhillon, Rana; Maher, Chris; Zadro, Joshua

---

VERSION 1 - REVIEW

**Reviewer**	**1**
**Name**	**Bhandutia , Amit**
**Affiliation**	**LSUHSC**
**Date**	**05-Aug-2025**
**COI**	**None**

The study represents the development process of decision aid for patients with degenerative cervical myelopathy. The authors interviewed and utilized questionnaires to query both health care professionals in addition to patients with degenerative cervical myelopathy. The study is unique in that patient considerations are used to improve the quality of the decision-making aid. This study which helps to raise awareness of DCM - consistent with the first research priority of AO Spine RECODE. This is especially important in taking patient considerations into mind. I personally enjoyed reading through the manuscript and will take these considerations into mind in developing my own patient decision aid.

**Reviewer**	**2**
**Name**	**Schoeller, Soraia**
**Affiliation**	**Universidade Federal de Santa Catarina**
**Date**	**25-Sep-2025**

COI None

Innovative theme. Rigorous and well described method. Results compatible with objectives and method. I suggest adding a paragraph about the importance of the patient's decision and this being central to multiprofessional treatment.

Reviewer 3

Name Uddin, Taslim

Affiliation Bangabandhu Sheikh Mujib Medical University, Physical Medicine and Rehabilitation

Date 06-Jan-2026

COI None

Thank you, authors, for developing this thoughtful and clinically meaningful manuscript. This decision aid for DCM is timely, and the authors have clearly invested substantial effort in stakeholder engagement. However, several aspects of the study design, methods description, statistical analysis, and manuscript text require clarification or strengthening.

Please find the comments below.

a) Clearly state the mixed-methods design. When was the study performed? Specify whether the quantitative part is descriptive, hypothesis-testing, or a pilot, and whether the qualitative part is thematic analysis, grounded theory-oriented, user-centered design evaluation, or framework analysis.

b) Clarify which characteristics guided the sampling, how many people were screened and excluded, whether saturation was assessed separately for patients and clinicians, and how “three consecutive participants” was applied in practice. Also, because the sample is imbalanced (for example, mostly female patients, mostly male clinicians, and many are already post-surgery), please discuss how this may bias the results. Please conceptually justify the sample size.

c)The current description of the analyses is too general. Please specify how Likert responses were handled, whether normality was tested before using means and SDs, whether confidence intervals were calculated, and how missing data were managed.

d) Please identify the primary quantitative outcomes (such as overall acceptability, usefulness, or decision difficulty) and clarify whether the results are exploratory.

e) I find the qualitative component of the mixed method study is central but needs more methodological detail, including how the coding framework was developed and refined, how reflexivity and researchers’ prior involvement were considered, whether participant validation was done on themes rather than just transcripts, and whether any qualitative

software was used in addition to Excel. A brief extract or coding tree would enhance credibility. Further, the paper should clearly explain whether qualitative findings informed the interpretation of questionnaire results, whether triangulation was used, and whether differences between clinician and patient perspectives were systematically explored.

f) Because there is no control group and only descriptive analysis was made, claims should be made to reflect acceptability and feasibility rather than impact.

As a a reader of the article, the storytelling was misguiding and confusing.

a) Throughout the manuscript, the terms “patient” and “people” are used interchangeably. For clarity, please specify who is a patient and who are people living with DCM (Page 6, Line - 6-7, 11-12, 22-23, 39-40, Page 7, Lines 4-6, and more)

b) Table-1: Employment Status; Page 16, Lines 26-31: Exact occupation was not provided, which is a very important risk factor for DCM development. I have read an article by Baucher G, et al. Risk factors for development of DCM: A review. https://pubmed.ncbi.nlm.nih.gov/34845577/

c) Table 2: Health Professionals (lines 28-30) and texts in (page 6, line 47, and in many places)—‘Rehabilitation Specialist’ and ‘Occupational Physician’—what’s the exact specialty?

d) Limitations: It appears all 10 participating countries are from high-income countries; it should be mentioned

Thank you.

VERSION 1 - AUTHOR RESPONSE

Reviewer #1:

Dr. Amit Bhandutia , LSUHSC

Comments to the Author:

The study represents the development process of decision aid for patients with degenerative cervical myelopathy. The authors interviewed and utilized questionnaires to query both health care professionals in addition to patients with degenerative cervical myelopathy. The study is unique in that patient considerations are used to improve the quality of the decision-making aid. This study which helps to raise awareness of DCM - consistent with the first research priority of AO Spine RECODE. This is especially important in taking patient considerations

into mind. I personally enjoyed reading through the manuscript and will take these considerations into mind in developing my own patient decision aid.

Comment 1:

Response

We thank the reviewer for their comments.

Reviewer #2:

Dr. Soraia Schoeller, Universidade Federal de Santa Catarina

Comments to the Author:

Innovative theme. Rigorous and well described method. Results compatible with objectives and method.

Comment 1:

I suggest adding a paragraph about the importance of the patient's decision and this being central to multiprofessional treatment.

Response

We have now added information on the importance of patient decision aids and multiprofessional treatment in the introduction and a paragraph in relation to DCM specifically in the discussion.

Introduction

Patient decision aids outline expected benefits and risks of management options by providing evidence-based information[21]. Patient decision aids aim to better align

treatment choices with patient’s personal values and in many cases promote multiprofessional involvement in treatment decisions[22].

Discussion

Our patient decision aid for people with DCM considering surgery could be a useful resource to promote continuity of care for people with DCM and align treatment with evidence-based guidelines. Patient decision aids have been shown to lead to large increases in patient knowledge, expectations of benefits and harms, and participation in making the decision[21]. Multiprofessional use of patient decision aids, including this DCM decision aid, can promote ‘shared understanding’ of decisions and introduce the concept of choice at the beginning and during consults with various health professionals during a patients’ journey[22]. Participant interviews supported this, suggesting that multiprofessional treatment is key for people with DCM and our patient decision aid is feasible to facilitate discussions with a variety of health professionals (e.g. general practitioners, surgeons, allied health clinicians).

Reviewer #3:

Dr. Taslim Uddin, Bangabandhu Sheikh Mujib Medical University

Comments to the Author:

Thank you, authors, for developing this thoughtful and clinically meaningful manuscript. This decision aid for DCM is timely, and the authors have clearly invested substantial effort in stakeholder engagement. However, several aspects of the study design, methods description, statistical analysis, and manuscript text require clarification or strengthening. Please find the comments below.

Comment 1:

a) Clearly state the mixed-methods design. When was the study performed? Specify whether the quantitative part is descriptive, hypothesis-testing, or a pilot, and whether the qualitative part is thematic analysis, grounded theory-oriented, user-centred design evaluation, or framework analysis.

Response

We have specified the qualitative and quantitative aspects of the study in line with these suggestions.

Abstract

Participants: Patient-participants and health professional-participants who manage people with degenerative cervical myelopathy were recruited through social media and the steering group's research and practice network. Quantitative questionnaires were used to gather baseline data, descriptive feedback, refine the decision aid, and assess its acceptability. Qualitative semi-structured interviews were conducted online to gather feedback on the decision aid and were analysed using reflexive thematic analysis.

Methods

2.4 Data analysis

Pre-interview and acceptability questionnaire responses were summarised using descriptive statistics including medians, interquartile ranges (IQR), counts and percentages). Due to a low sample size and data that were not normally distributed,

we reported median (IQR) and did not calculate confidence intervals. No inferential statistics were reported as our analysis of quantitative data was purely descriptive.

All qualitative aspects of this study were reported according to the 32-item Consolidated Criteria for Reporting Qualitative Research (COREQ) checklist (supplementary file 9)[42]. All interview data were analysed using thematic analysis; a method for identifying, analysing and reporting patterns within data[43]. Grounded theory using an inductive approach underpinned how data were collected and analysed[44]. Two researchers (AG and SM) independently recorded initial themes, familiarised themselves with the interviews via audio recordings or transcripts and developed a framework to organise concepts into broader themes and subthemes in Microsoft Excel. No qualitative software was used in addition to Excel due to the pragmatic and simplistic nature of our analysis (i.e., analysing feedback on the decision aid). The mapping of themes and subthemes (figure 1) was achieved through multiple iterative cycles as new data emerged. Any disagreements were discussed and resolved with a third author (JZ). The decision aid was continually updated before new interviews and circulated to the study authors to reach consensus. Consensus was determined by the majority of the study authors agreeing with proposed changes. No further interviews were conducted once data saturation was achieved (i.e., no new feedback emerged that would lead to a new theme or subtheme from three consecutive participants) and participants had an overall positive impression of the decision aid. Saturation was assessed separately for patient-participants and health professional-participants. Triangulation[45] was used to inform the interpretation of results by considering whether the quantitative results were consistent with the overall impression of the decision aid by participants during interviews.

Results

3.2 Participant characteristics and decision aid acceptability

A total of 32 initial interviews were conducted; 19 patient-participants and 13 health professional-participants who manage people with DCM (table 1 and 2). Interview participants were selected from 54 people with DCM and 16 health professionals who completed the per-interview survey. Two male participants attended an interview but withdrew before the interview began because we were unable to offer any compensation for their time. No participant was excluded. Interviews were conducted from March to September 2024. The results from the acceptability questionnaires are presented in table 3 and figure 2.

Comment 2:

b) Clarify which characteristics guided the sampling, how many people were screened and excluded, whether saturation was assessed separately for patients and clinicians, and how "three consecutive participants" was applied in practice. Also, because the sample is imbalanced (for example, mostly female patients, mostly male clinicians, and many are already post-surgery), please discuss how this may bias the results. Please conceptually justify the sample size.

Response

We have conceptually justified the sample size and amended the description of purposive sampling to provide examples of characteristics that guided sampling. We have added further description of how data saturation using "three consecutive participants" was applied. We have added the number of people screened and that no

participant was excluded to the results section. We have also highlighted that the imbalanced sample is a limitation in the strengths and limitations section of the manuscript.

Methods

2.21 Sample size

A sample size of approximately 30 participants was identified as likely sufficient based on previous mixed-methods studies describing the development of patient decision aids[24,25,38]. This was later confirmed by data saturation.

2.31 Pre interview questionnaires

Pre-interview questionnaires were completed electronically by potential participants to gather baseline information. Collected data on demographics (e.g., age, gender, education), clinical information (e.g., had surgery for DCM) and professional background (table 1 and 2) were used to purposively sample participants where possible.

2.4 Data analysis

Pre-interview and acceptability questionnaire responses were summarised using descriptive statistics including: medians, interquartile ranges (IQR), counts and percentages. Due to a low sample size and data that were not normally distributed, we reported median (IQR) and did not calculate confidence intervals. No inferential statistics were reported as our analysis of quantitative data was purely descriptive.

All qualitative aspects of this study were reported according to the 32-item Consolidated Criteria for Reporting Qualitative Research (COREQ) checklist (supplementary file 9)[42].

All interview data were analysed using thematic analysis; a method for identifying, analysing and reporting patterns within data[43]. Grounded theory using an inductive approach underpinned how data were collected and analysed[44]. Two researchers (AG and SM) independently recorded initial themes, familiarised themselves with the interviews via audio recordings or transcripts and developed a framework to organise concepts into broader themes and subthemes in Microsoft Excel. No qualitative software was used in addition to Excel due to the pragmatic and simplistic nature of our analysis (i.e., analysing feedback on the decision aid). The mapping of themes and subthemes (figure 1) was achieved through multiple iterative cycles as new data emerged. Any disagreements were discussed and resolved with a third author (JZ). The decision aid was continually updated before new interviews and circulated to the study authors to reach consensus. Consensus was determined by the majority of the study authors agreeing with proposed changes. No further interviews were conducted once data saturation was achieved (i.e., no new feedback emerged that would lead to a new theme or subtheme from three consecutive participants) and participants had an overall positive impression of the decision aid. Saturation was assessed separately for patient-participants and health professional-participants. Triangulation[45] was used to inform the interpretation of results by considering whether the quantitative results were consistent with the overall impression of the decision aid by participants during interviews.

Results

3.2 Participant characteristics and decision aid acceptability

A total of 32 initial interviews were conducted; 19 patient-participants and 13 health professional-participants who manage people with DCM (table 1 and 2). Interview participants were selected from 54 people with DCM and 16 health professionals who completed the per-interview survey. Two male participants attend an interview but withdrew before the interview began because we were unable to offer any compensation for their time. No participant was excluded. Interviews were conducted from March to September 2024. The results from the acceptability questionnaires are presented in table 3 and figure 2.

Discussion

4.3 Strengths and limitations

Limitations include challenges with recruitment. For example, despite using purposive sampling, most patient-participants were females (89%), most health professional-participants were males (85%) and most patient-participants had undergone surgery (79%).

Comment 3:

c) The current description of the analyses is too general. Please specify how Likert responses were handled, whether normality was tested before using means and SDs, whether confidence intervals were calculated, and how missing data were managed.

Response

The data were not normally distributed, so we have now reported values using medians (IQR). We did not conduct statistical tests as the quantitative aspect of this

study was descriptive. Since we did not conduct statistical tests and our data was not normally distributed, we did not calculate confidence intervals. Any missing data are noted in the table footnotes.

Methods

2.4 Data analysis

Pre-interview and acceptability questionnaire responses were summarised using descriptive statistics including medians, interquartile ranges (IQR), counts and percentages. Due to a low sample size and data that were not normally distributed, we reported median (IQR) and did not calculate confidence intervals. No inferential statistics were reported as our analysis of quantitative data was purely descriptive.

Comment 4:

d) Please identify the primary quantitative outcomes (such as overall acceptability, usefulness, or decision difficulty) and clarify whether the results are exploratory.

Response

We have identified the primary quantitative outcome and outlined that results were descriptive and not exploratory.

Methods

2.3 Data collection

We collected quantitative and qualitative data. The data collection process involved pre-interview questionnaires (supplementary files 2 and 3), semi-structured interviews (supplementary file 4 and 5), and acceptability questionnaires (supplementary file 6

and 7) for patient- and health professional-participants. The primary quantitative outcome was the overall acceptability of the patient decision aid as assessed by rating the content, length, amount and balance of information, potential use of decision aid, impact on decision difficulty and implementation (Table 3 and Figure 2).

2.4 Data analysis

Pre-interview and acceptability questionnaire responses were summarised using descriptive statistics including medians, interquartile ranges (IQR), counts and percentages. Due to a low sample size and data that were not normally distributed, we reported median (IQR) and did not calculate confidence intervals. No inferential statistics were reported as our analysis of quantitative data was purely descriptive.

Comment 5:

e) I find the qualitative component of the mixed method study is central but needs more methodological detail, including how the coding framework was developed and refined, how reflexivity and researchers' prior involvement were considered, whether participant validation was done on themes rather than just transcripts, and whether any qualitative software was used in addition to Excel. A brief extract or coding tree would enhance credibility. Further, the paper should clearly explain whether qualitative findings informed the interpretation of questionnaire results, whether triangulation was used, and whether differences between clinician and patient perspectives were systematically explored.

Response

We have included how the coding framework was developed and refined with an additional figure (figure 3) showing an example coding tree. We have also outlined

that triangulation was used and how data from participants with DCM and health professionals was used.

Methods

3.3 Feedback for each section of the decision aid

Most suggestions from participants were incorporated into the decision aid but some were not possible to implement as they conflicted with the best available evidence or other participants' feedback. Online supplementary file 13 outlines the feedback we did not incorporate in the decision aid and our justification for this. Figure 3 provides an example of the how coding was used to develop themes and subthemes.

Figure 3: Formation of subthemes and themes.

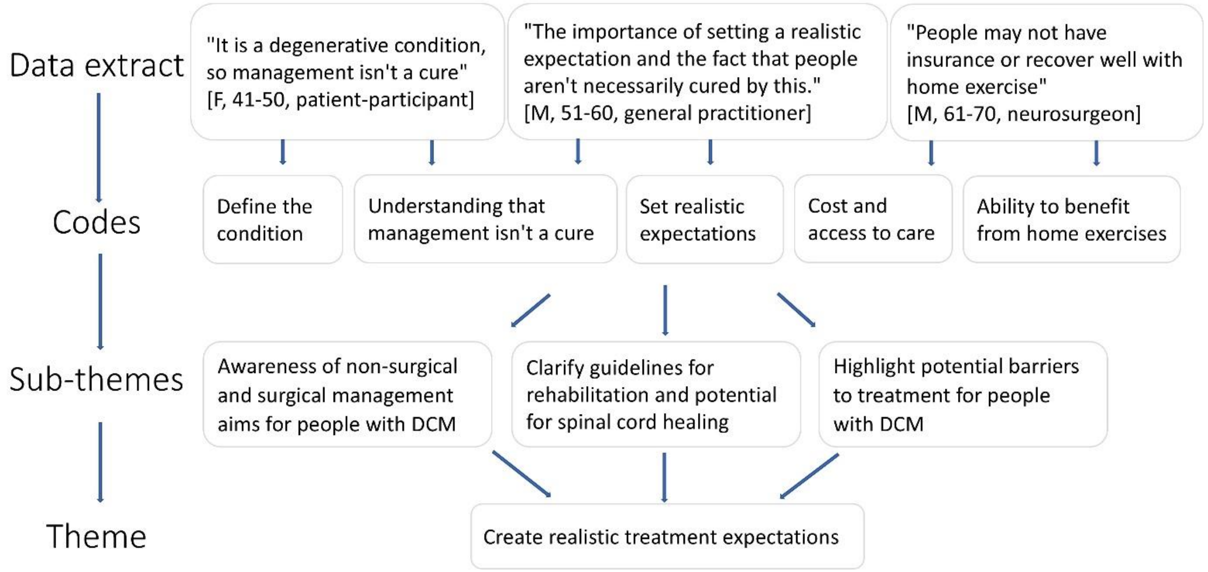

Methods

2.4 Data analysis

Pre-interview and acceptability questionnaire responses were summarised using descriptive statistics including medians, interquartile ranges (IQR), counts and percentages. Due to a low sample size and data that were not normally distributed, we reported median (IQR) and did not calculate confidence intervals. No inferential statistics were reported as our analysis of quantitative data was purely descriptive.

All qualitative aspects of this study were reported according to the 32-item Consolidated Criteria for Reporting Qualitative Research (COREQ) checklist (supplementary file 9)[42]. All interview data were analysed using thematic analysis; a method for identifying, analysing and reporting patterns within data[43]. Grounded theory using an inductive approach underpinned how data were collected and analysed[44]. Two researchers (AG and SM) independently recorded initial themes, familiarised themselves with the interviews via audio recordings or transcripts and developed a framework to organise concepts into broader themes and subthemes in Microsoft Excel. No qualitative software was used in addition to Excel due to the pragmatic and simplistic nature of our analysis (i.e., analysing feedback on the decision aid). The mapping of themes and subthemes (figure 1) was achieved through multiple iterative cycles as new data emerged. Any disagreements were discussed and resolved with a third author (JZ). The decision aid was continually updated before new interviews and circulated to the study authors to reach consensus. Consensus was determined by the majority of the study authors agreeing with proposed changes. No further interviews were conducted once data saturation was achieved (i.e., no new feedback emerged that would lead to a new theme or subtheme from three consecutive participants) and participants had an overall positive impression of the decision aid. Saturation was assessed separately for patient-participants and health professional-

participants. Triangulation[45] was used to inform the interpretation of results by considering whether the quantitative results were consistent with the overall impression of the decision aid by participants during interviews.

Comment 6:

f) Because there is no control group and only descriptive analysis was made, claims should be made to reflect acceptability and feasibility rather than impact.

Response

We have updated the manuscript language to focus on acceptability and feasibility rather than impact throughout.

Discussion

Future acceptability of our decision aid may be facilitated by enhancing visual appeal, simplicity of design and ease of use. For example, 89% of patient-participants who completed the acceptability questionnaire said our decision aid would have made their decision easier (table 2) and all health professional-participants 'strongly agreed' or 'agreed' it would be easy to use and understand. Actioning feedback to facilitate shared decision-making and reduce complexity (e.g. an external link to the mJOA) may mean the aid is more feasible to be integrated into routine care than other more complex educational tools[22]. Incorporating plain language, bullet points, and appropriate visuals could also facilitate acceptability[49]. Dot point questions about quality of life and pain included take into account the 'ceiling-effect' of the mJOA for people with mild DCM[50].

The decision aid includes numeric estimates of benefits and harms, links to social groups, and a statement that research on neural plasticity and rehabilitation is ongoing. Including these features may reduce potential anxiety and improve quality of life (e.g. mood and motivation) for people living with DCM irrespective of their situation[53].

Our patient decision aid for people with DCM considering surgery could be a useful resource to promote continuity of care for people with DCM and align treatment with evidence-based guidelines. Patient decision aids have been shown to lead to large increases in patient knowledge, expectations of benefits and harms, and participation in making the decision[21]. Multiprofessional use of patient decision aids, including this DCM decision aid, can promote 'shared understanding' of decisions and introduce the concept of choice at the beginning and during consultations with various health professionals during a patients' journey[22]. Participant interviews supported this, suggesting that multiprofessional treatment is key for people with DCM and our patient decision aid is feasible to facilitate discussions with a variety of health professionals (e.g. general practitioners, surgeons, allied health).

4.3 Strengths and limitations

Limitations include challenges with recruitment. For example, despite using purposive sampling, most patient-participants were females (89%), most health professional-participants were males (85%) and most patient-participants had undergone surgery (79%). Health professional-participants were from eight different professions and eight different countries of training which has advantages and disadvantages. Recruiting from diverse settings increases the generalisability of our

study results for potential use of the tool with different health professionals in several countries but reduces region-specificity and may limit acceptability to low-to-middle income countries. Nonetheless, we prioritised developing an acceptable decision aid for use in different countries to increase reach, promote earlier diagnosis and encourage evidence-based management for people with DCM globally. Future research should include the exact occupation of patient-participants as this appears to be an important risk factor for DCM development based on previous research[54]. The decision aid and other future resources (e.g. supplementary video) will need to be translated to other languages for people from non-English speaking backgrounds. The decision aid appears to be acceptable but there is still uncertainty in the existing evidence comparing surgery to non-surgical management and a need to consider individualised management.

Conclusion

Our decision aid for people with DCM was rated as an acceptable tool by patient-participants and health professional-participants involved in their management. This decision aid is feasible for use by clinicians and people diagnosed with DCM to help with shared decision making. The tool is likely acceptable and feasible for people with mild DCM, given surgical management may not yet be mandatory, and a variety of non-surgical management options are still available. During the study, our interviews reinforced the need to increase awareness of DCM, improve monitoring of symptoms and ensure ease of access to the decision aid to optimise global impact. We encourage the use of the decision aid in daily clinical practice, while a larger study evaluating the potential benefits of the decision aid is being planned.

Comment 7:

As a reader of the article, the storytelling was misguiding and confusing.

a) Throughout the manuscript, the terms “patient” and “people” are used interchangeably. For clarity, please specify who is a patient and who are people living with DCM (Page 6, Line -6-7, 11-12, 22-23, 39-40, Page 7, Lines 4-6, and more)

Response

We thank the reviewer for the suggestions, and we have updated the manuscript to use the term patient-participants to describe people with DCM who participated in the study and health professional-participants for health professionals who participated in the study.

Comment 8:

b) Table-1: Employment Status; Page 16, Lines 26-31: Exact occupation was not provided, which is a very important risk factor for DCM development. I have read an article by Baucher G, et al. Risk factors for development of DCM: A review.

https://pubmed.ncbi.nlm.nih.gov/34845577/

Response

We collected employment status but did not collect data on occupation. We have now included this as a limitation with the suggested reference.

Discussion

4.3 Strengths and limitations

Future research should include the occupation of patient-participants as this appears to be an important risk factor for DCM development based on previous research[54].

Comment 9:

c) Table 2: Health Professionals (lines 28-30) and texts in (page 6, line 47, and in many places)—'Rehabilitation Specialist' and 'Occupational Physician'—what's the exact specialty?

Response

We have provided a more detailed description of these occupations in the manuscript and provided a definition in the footnotes under Table 2.

Abstract

Results: We conducted 32 interviews; 19 patient-participants and 13 health professional-participants who manage people with degenerative cervical myelopathy (neurosurgeons, neurologists, physiotherapists, orthopaedic surgeon, general practitioner, rehabilitation and pain specialist and consultant occupational physician and chiropractor/researcher). Participants were from 10 countries (Australia, Canada, Cyprus, Germany, Ireland, New Zealand, Sweden, Switzerland, United Kingdom and USA). Most participants rated the decision aid's acceptability as good-to-excellent and agreed with most aspects of the decision aid (e.g., defining degenerative cervical myelopathy, management recommendations, potential benefits and harms, questions to consider asking a health professional).

Table 2: Characteristics of health professional-participants who manage people with degenerative cervical myelopathy

Characteristics		Health professional-participants (n=13)
**Age in years, mean (SD)**		49 (12)
**Female**		2 (15%)
**Country of health professional training**[a]	Australia	4 (27%)
	United Kingdom	3 (20%)
	Canada	2 (13%)
	USA	2 (13%)
	Germany	1 (7%)
	India	1 (7%)
	New Zealand	1 (7%)
	Switzerland	1 (7%)
**Professional background**	Neurosurgeon	4 (31%)
	Neurologist	2 (15%)
	Physiotherapist	2 (15%)
	Orthopaedic surgeon	1 (8%)
	General practitioner	1 (8%)
	Rehabilitation and pain specialist[b]	1 (8%)
	Consultant occupational physician[c]	1 (8%)
	Chiropractor/researcher	1 (8%)
**Years of experience, mean (SD)**		18 (11)
**Work setting**	Private practice	4 (31%)
	Private hospital	0 (0%)
	Public hospital	7 (54%)
	Other	2 (15%)
**Average number of people with DCM managed per year**	5-10	3 (23%)
	11-20	1 (8%)
	21-30	3 (23%)
	31-40	1 (8%)
	>40	5 (38%)
**Median (IQR) percentage of people diagnosed with DCM they would advise to have surgery**		60% (40% to 70%)
**Median (IQR) percentage of people diagnosed with mild DCM they would advise to have surgery**		20% (10% to 45%)

N, number of participants. DCM, Degenerative Cervical Myelopathy. SD, Standard Deviation. USA, United States of America. IQR, Interquartile range. [a]All health professional-participants were practicing in their country of training at the time of interviews. One health professional-participant practicing in Australia also trained in India and the United Kingdom. [b]Rehabilitation and pain specialists specialise in spinal injuries, regional pain and the management of acute and persistent pain. [c]Consultant occupational physicians specialise in the management and rehabilitation of injured workers and general management of spine conditions.

Comment 10:

d) Limitations: It appears all 10 participating countries are from high-income countries; it

should be mentioned

Response

We have included that the decision aid may have limited acceptability in low-to-middle income countries.

Methods

4.3 Strengths and Limitations

Recruiting from diverse settings increases the generalisability of our study results for potential use of the tool with different health professionals in several countries but reduces region-specificity and may limit acceptability to low-to-middle income countries.

VERSION 2 - REVIEW

**Reviewer**	**3**
**Name**	**Uddin, Taslim**
**Affiliation**	**Bangabandhu Sheikh Mujib Medical University, Physical Medicine and Rehabilitation**
**Date**	**07-Feb-2026**
**COI**	

Major reviewer concerns were addressed; however, the following reviewer observations required attention. a) Section 2.4: Simultaneous claims of thematic analysis and grounded theory underpinning appear confusing; we suggest clarifying grounded theory as an influence rather than a formal methodology, or removing it to avoid methodological overreach. Please check the reference number 44 and others

b) Figure 3 improves transparency. Reflexivity, i.e., researcher positioning, is still minimally addressed, and there is no clear statement on participant validation of themes. Differences between clinician and patient perspectives are acknowledged but not systematically analyzed. Improvement is required with a brief reflexivity sentence and a note on whether theme validation was or was not performed. Thank you.

VERSION 2 - AUTHOR RESPONSE

Reviewer: 3

Dr. Taslim Uddin, Bangabandhu Sheikh Mujib Medical University

Comments to the Author:

Major reviewer concerns were addressed; however, the following reviewer observations required attention.

Comment 1:

a) Section 2.4: Simultaneous claims of thematic analysis and grounded theory underpinning appear confusing; we suggest clarifying grounded theory as an influence rather than a formal methodology, or removing it to avoid methodological overreach. Please check the reference number 44 and others

Response

We thank the reviewer for their comments. On further consideration we have removed mention of grounded theory to avoid confusion. We have removed reference 44 and reviewed all other references.

Methods

2.4 Data analysis

All qualitative aspects of this study were reported according to the 32-item Consolidated Criteria for Reporting Qualitative Research (COREQ) checklist (supplementary file 9)[42]. All interview data were analysed using thematic analysis; a method for identifying, analysing and reporting patterns within data[43]. The positionality of the interviewer and authorship team aligned with the best available evidence highlighting that people with mild DCM may not need surgery and should be engage in shared decision-making, and people with moderate or severe DCM

require timely surgery[17,19]. Two physiotherapist researchers (AG and SM) independently recorded initial themes, familiarised themselves with the interviews via audio recordings or transcripts and developed a framework to organise concepts into broader themes and subthemes in Microsoft Excel. No qualitative software was used in addition to Excel due to the pragmatic and simplistic nature of our analysis (i.e., analysing feedback on the decision aid). The mapping of themes and subthemes (figure 1) was achieved using a descriptive approach and multiple iterative cycles as new data emerged. Interview themes were not interpreted or validated by participants to protect the legitimacy of the authorship's team interpretation as a central analytic process and allow themes to reflect patterns across the dataset rather than individual preferences. Any disagreements were discussed and resolved with a third author (JZ). The decision aid was continually updated before new interviews and circulated to the study authors to reach consensus. Consensus was determined by the majority of the study authors agreeing with proposed changes. No further interviews were conducted once data saturation was achieved (i.e., no new feedback emerged that would lead to a new theme or subtheme from three consecutive participants) and participants had an overall positive impression of the decision aid. Saturation was assessed separately for patient-participants and health professional-participants. Triangulation[44] was used to inform the interpretation of results by considering whether the quantitative results were consistent with the overall impression of the decision aid by participants during interviews.

Comment 2:

b) Figure 3 improves transparency. Reflexivity, i.e., researcher positioning, is still minimally addressed, and there is no clear statement on participant validation of themes. Differences

between clinician and patient perspectives are acknowledged but not systematically analyzed. Improvement is required with a brief reflexivity sentence and a note on whether theme validation was or was not performed. Thank you.

Response

We thank the reviewer for these comments. We have added a reflexivity statement on researcher positioning in the methods section, highlighting that the positionality of the interviewer and authorship team was that people with mild DCM may not need surgery and should be engage in shared decision-making, and people with moderate or severe DCM require timely surgery. We have provided a statement that theme validation was not performed and provided some reasons for this approach. (see above)

Systematically analysing differences between clinician- and patient-perspectives was not an aim of this study, as we wanted to develop a tool that is acceptable and feasible to use in clinical settings during a discussion between patients and their clinicians. However, we have now expanded on the differences between clinician and patient feedback during interviews and highlighted where to find this data (Supplementary file 13).

Results

3.3 Feedback for each section of the decision aid

Most suggestions from participants were incorporated into the decision aid but some were not possible to implement as they conflicted with the best available evidence or other participants' feedback. Patient- and health professional-participant feedback was analysed separately but presented in a combined summary below. Online supplementary file 13

outlines instances where patient- and health professional feedback did or did not align, why some feedback was not incorporate in the decision aid and our justification for this. For example, patient- and health professional participants agreed access and affordability to healthcare are barriers to optimal treatment but had different views on how the mJOA tool should be presented in the decision aid. Patient-participants didn't think it was necessary to feature the mJOA prominently while health professional-participants thought the opposite. Figure 3 provides an example of the how coding was used to develop themes and subthemes.
